# Multicellular immune dynamics implicate PIM1 as a potential therapeutic target for uveitis

**DOI:** 10.1038/s41467-022-33502-7

**Published:** 2022-10-04

**Authors:** He Li, Lihui Xie, Lei Zhu, Zhaohuai Li, Rong Wang, Xiuxing Liu, Zhaohao Huang, Binyao Chen, Yuehan Gao, Lai Wei, Chang He, Rong Ju, Yizhi Liu, Xialin Liu, Yingfeng Zheng, Wenru Su

**Affiliations:** 1 State Key Laboratory of Ophthalmology, Zhongshan Ophthalmic Center, Sun Yat-sen University, Guangdong Provincial Key Laboratory of Ophthalmology and Visual Science, Guangzhou, 510060 China; 2 Research Unit of Ocular Development and Regeneration, Chinese Academy of Medical Sciences, Beijing, 100085 China

**Keywords:** Autoimmunity, Autoimmune diseases, Autoimmune diseases, Gene regulation in immune cells, Lymphocytes

## Abstract

Uveitis is a severe autoimmune disease, and a common cause of blindness; however, its individual cellular dynamics and pathogenic mechanism remain poorly understood. Herein, by performing single-cell RNA sequencing (scRNA-seq) on experimental autoimmune uveitis (EAU), we identify disease-associated alterations in cell composition and transcriptional regulation as the disease progressed, as well as a disease-related molecule, PIM1. Inhibiting PIM1 reduces the Th17 cell proportion and increases the Treg cell proportion, likely due to regulation of PIM1 to the protein kinase B (AKT)/Forkhead box O1 (FOXO1) pathway. Moreover, inhibiting PIM1 reduces Th17 cell pathogenicity and reduces plasma cell differentiation. Importantly, the upregulation of PIM1 in CD4^+^ T cells and plasma cells is conserved in a human uveitis, Vogt-Koyanagi-Harada disease (VKH), and inhibition of PIM1 reduces CD4^+^ T and B cell expansion. Collectively, a dynamic immune cellular atlas during uveitis is developed and implicate that PIM1 may be a potential therapeutic target for VKH.

## Abstract (web summary)

Uveitis is a complex autoimmune inflammatory disease of the eye and defining molecules involved is a priority. Here the authors use scRNA sequencing in mouse experimental autoimmune uveitis (EAU) and show PIM1 promotes the imbalance of Th17 and Treg cells, and find elevated PIM-1 in human uveitis disease.

## Introduction

Uveitis, an autoimmune eye disease involving the ciliary body, vitreous, choroid, and retina^1^, is a leading cause of blindness and characterized by recurring retinal and uveal inflammation^2^. Uveitis is also a central nervous system (CNS) autoimmune disease which differ from autoimmune diseases of other organs or tissues due to the relative immune privilege of the CNS^3,4^. After diagnosis of uveitis, treatment with systemic and topical glucocorticoids with or without immunosuppressive agents is required to prevent irreversible vision loss^5^. Glucocorticoids, together with immunosuppressive drugs, cause various side effects and show limited efficacy^6^. Studies of the underlying pathological mechanisms are needed to develop specific, safe, and effective treatment regimens for uveitis.

Abnormalities in various immune cells are critical contributors to autoimmune destruction in uveitis^7–10^. CD4^+^ T cells, especially regulatory (Treg) and effector (e.g., T helper (Th)−1 and Th17 cells) T cells, are considered pivotal in the pathogenesis of uveitis and its classic animal model, experimental autoimmune uveitis (EAU)^7,8,10^. Among them, Th17 cells dominate EAU pathogenesis^10,11^. Transfer of autoreactive Th17 cells can induce EAU in naïve mice^10,12^. In addition, increasing evidence supports the disease-promoting effect of B cells in EAU, although less certain than that of T cells ^9^. Nevertheless, a clear picture of the regulation of CD4^+^ T cells and B cells and the changes associated with the progression of uveitis is lacking. A dynamic atlas of immune dysfunction is needed to obtain a deeper understanding of the pathological mechanism of uveitis.

Lymph nodes (LN) are critical for autoimmune processes^13,14^. As the sites of interplay among various immune cells, especially T cells, B cells, and dendritic cells (DC), LNs facilitate antigen presentation, immune cell activation, and autoimmune response initiatio^15^. Cervical draining LNs (CDLN) are the major LNs promoting efficient drainage of macromolecules and immune cells from the CNS^16^. Thus, CDLNs might be the production site for autoreactive T cells and B cells that target CNS autoantigens. Removing CDLNs before the induction of experimental autoimmune encephalomyelitis (EAE), a classic animal model of multiple sclerosis that shares abundant similarities in the pathogenic mechanisms with EAU^17^, significantly reduces disease severity^13^.

In this work, we construct a comprehensive single-cell atlas of immune cells from CDLNs of EAU mice at various time points (day 0, 7, 14, and 21), depicting alterations in immune cell compositions and gene expression as the disease progressed. In particular, our single-cell data implicates that PIM1 is preferentially upregulated in Th17 cells, Th1 cells, Treg cells, and plasma cells (PC) during EAU. Repressing PIM1 reduces the imbalance of Th17/Treg cell and PC differentiation. Moreover, inhibiting PIM1 reduces Th17 pathogenicity. Furthermore, our study verifies the upregulation of PIM1 in CD4^+^ T cells and PCs in a human uveitis, Vogt-Koyanagi-Harada disease (VKH). Inhibiting PIM1 also suppresses the proliferation of CD4^+^ T cells and B cells in patients with VKH. Our data suggest that targeting PIM1 kinase may be a potential therapy for VKH.

## Results

### Dynamic changes in CDLN cells during EAU

**Fig. 1:**
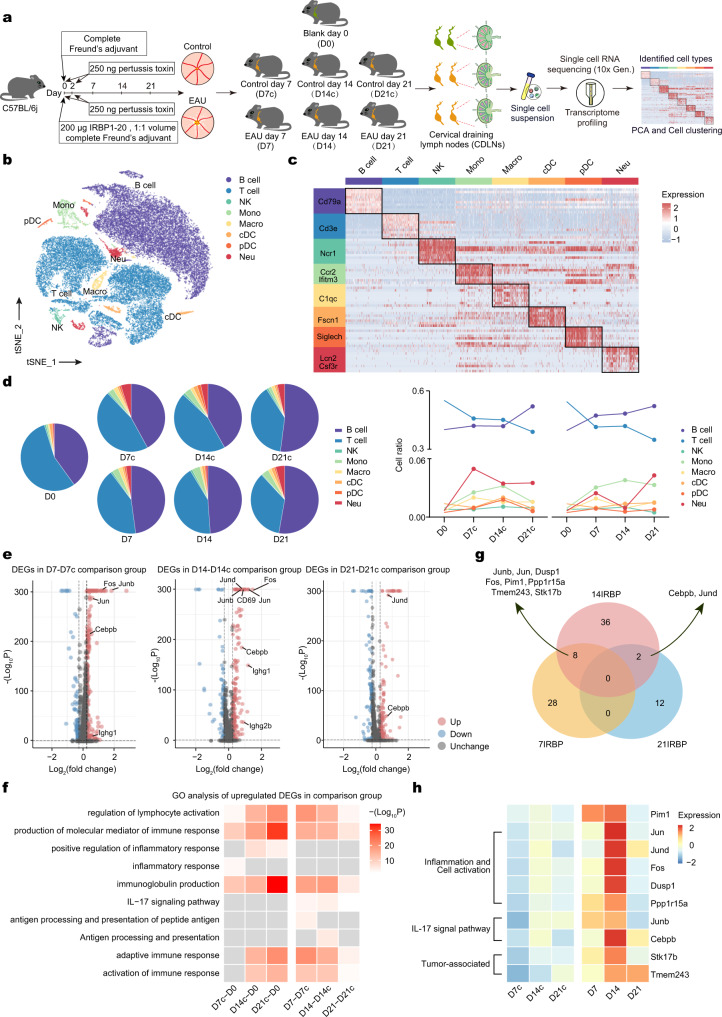
Study design and scRNA-seq analysis of EAU in multiple time points. **a** Schematic of the experimental design for single-cell RNA sequencing analysis. CDLN cells were harvested from control groups or EAU groups at different time points. Day 0 group (D0) and each EAU groups (D7, D14, D21) included two samples, while each control group (D7c, D14c, D21c) include one sample. Each sample included three mice. **b** t-Distributed stochastic neighbor embedding (t-SNE) clustering of CDLN cells from all mice groups. **c** Heatmap showing scaled expression of discriminative gene sets for major immune cell types in CDLNs from all mice groups. **d** Pie charts and line charts showing the percentages of the major immune cell types in control or EAU groups at different time points. **e** Volcano plot showing upregulated and downregulated DEGs of total immune cells in the EAU/control comparison groups at different time points. Red and blue dots indicate upregulated and downregulated DEGs in EAU groups compared to control groups, respectively. Significance was determined using “FindMarkers” functions of Seurat package with Wilcoxon Rank Sum test and adjusted by Bonferroni correction. **f** Heatmap showing representative GO terms and KEGG pathways enriched in upregulated DEGs of total immune cells in the control/day 0 comparison groups and EAU/control comparison groups at different time points. Significance was calculated based on the accumulative hypergeometric distribution by Metascape webtool. **g** Venn diagrams showing the number of upregulated DEGs in EAU groups compared to control groups without those in control groups compared to day 0 group at different time points. **h** Heatmap of the relative expression of the ten DEGs annotated in (G) in control and EAU groups at different time points.

To investigate the dynamic changes in the immune cellular response during EAU, we established the EAU model by injecting mice with a commonly used uveitogenic retinal protein, IRBP_1–20_^18^, along with complete Freund’s adjuvant and pertussis toxin. To exclude simple immunization- or manipulation-related effects induced by the reagents, mice in the control group were injected with complete Freund’s adjuvant and pertussis toxin only (Fig. 1a). In mice immunized with IRBP_1–20_, EAU developed on day 7 and the symptoms worsened on days 14 and 21 (day 7, 14, and 21 EAU groups). Mice sacrificed on day 0 before injection (defined as the day 0 group) and on days 7, 14, and 21 from the control group (days 7, 14, and 21 control groups) did not exhibit EAU symptoms (Supplementary Fig. 1a, b). Single-cell suspensions were obtained from mouse CDLNs from the above groups and translated into barcoded single cell RNA sequencing (scRNA-seq) libraries for subsequent research (Fig. 1a).

Eight major immune cell types, including B cells, T cells, natural killer (NK) cells, monocytes (Mono), macrophages (Macro), classical dendritic cells (cDC), plasmacytoid dendritic cells (pDC), and neutrophils (Neu) were identified in CDLNs according to classical lineage markers (Fig. 1b, c; Supplementary Fig. 1c, d). T cells and B cells were dominant among CDLN cells; myeloid cells formed a relatively small ratio (Fig. 1d). Cell proportions showed similar variation trends in the EAU and control groups (Fig. 1d).

To capture the dynamics of the global gene signature of EAU, we conducted differentially expressed gene (DEG) analysis between control groups and the day 0 group, and between EAU and control groups on corresponding days. Control groups showed upregulated inflammatory (*S100a8* and *S100a9*) and immunoglobulin production-related (*Ighg1* and *Ighg2b*) genes compared to the day 0 group (Supplementary Fig. 1e). Compared to control groups, EAU groups showed upregulated genes related to cell activation (*Fos*, *Jun*, *Jund*, and *CD69*), IL-17 signaling (*Cebpd* and *Junb*), and immunoglobulin production (*Ighg1* and *Ighg2b*) on different days (Fig. 1e). Subsequent GO analysis showed that pathways related to lymphocyte activation, immune response, and immunoglobulin production were enriched in the control groups compared to the day 0 group. These pathways were further enriched in all EAU groups compared with their corresponding control groups (Fig. 1f). Moreover, the IL-17 signaling pathway was uniquely enriched in EAU groups (Fig. 1f). To further investigate genes exclusively upregulated in EAU groups, we removed upregulated DEGs identified in control groups vs. the day 0 group comparison and constructed a Venn diagram (Fig. 1g). Ten genes were exclusively upregulated in EAU groups at two consecutive time points (Fig. 1g, h). Among them, *Jun*, *Jund*, *Fos*, *Dusp1*, and *Ppp1r15a* are related to inflammation and cell activation (Fig. 1h). *Junb* and *Cebpb* are related to the IL-17 signaling pathway (Fig. 1h). *Stk17b*, *Tmem243*, and *Pim1* are associated with tumors (Fig. 1h). Intriguingly, PIM1 is reportedly involved in tumor immunology^19^. The abnormal upregulation of this molecule in EAU aroused our interest.

Next, we explored the dynamic transcriptional changes of eight major immune cell types during EAU. In control groups, neutrophils and monocytes showed the largest number of DEGs on day 14 compared to day 0 (Supplementary Fig. 1f). In EAU groups, these two immune cell types exhibited the highest number of DEGs on day 7 compared to control groups (Supplementary Fig. 1f). On days 14 and 21, B cells and T cells had higher numbers of DEGs than myeloid cells in EAU groups (Supplementary Fig. 1f). We then conducted GO analysis to annotate the biological significance of these DEGs. In T cells in control groups, DEGs were enriched in pathways related to inflammation and cytokine signaling compared to the day 0 group, whereas the IL-17 signaling, leukocyte differentiation, and T cell activation pathways were enriched in EAU groups (Supplementary Fig. 2a). In B cells, pathways related to immunoglobulin production and B cell activation were enriched in control groups compared to the day 0 group. These pathways were further enriched in all EAU groups compared to their corresponding control groups (Supplementary Fig. 2b). Other transcriptional changes in EAU groups included enriched pathways annotated as the “TNF signaling pathway” in NK cells, “interleukin-1 beta production” in monocytes, “adaptive immune system” in macrophages, “antigen processing and presentation” in DCs, as well as “neutrophil degranulation” in neutrophils (Supplementary Fig. 2c–h). Generally, enrichment of the above pathways was more evident on days 7 and 14 compared to day 21 in EAU groups (Supplementary Fig. 2a–h), which might suggest acute inflammation in the first 2 weeks.

The above findings showed dynamic changes in the proportion and transcription of the major immune cell types in EAU compared to control groups which represented a condition affected by immunization- or manipulation-related effects.

### Dynamic changes and upregulation of Pim1 in T cell subsets during EAU

**Fig. 2:**
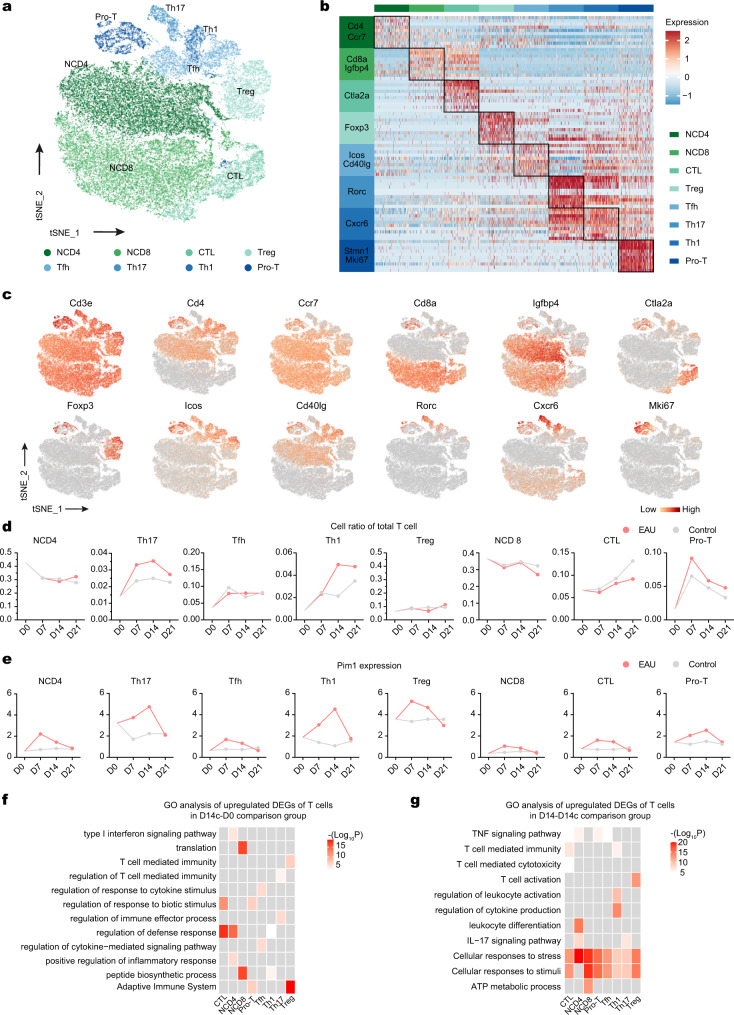
scRNA-seq analysis of the dynamic changes in T cell subsets. **a** t-SNE plots of T cell subsets from all mice groups. **b** Heatmap showing scaled expression of discriminative gene sets for T cell subsets from all mice groups. **c** t-SNE plots of canonical markers for T cell subsets from all mice groups. **d** Line charts showing the proportion of T cell subsets in total T cells from EAU and control groups at different time points. **e** Line charts showing the mean expression of Pim1 in T cell subsets from EAU and control groups at different time points. **f** Heatmap showing representative GO terms and KEGG pathways enriched in upregulated DEGs of the T cell subsets in the D14c/D0 comparison groups at different time points. Significance was calculated based on the accumulative hypergeometric distribution by Metascape webtool. **g** Heatmap showing representative GO terms and KEGG pathways enriched in upregulated DEGs of the T cell subsets in the D14/D14c comparison groups at different time points. Significance was calculated based on the accumulative hypergeometric distribution by Metascape webtool.

Considering the vital function of T cells in EAU pathogenesis, T cells were re-clustered into eight subsets including naïve CD4^+^ T (NCD4) cells, naïve CD8^+^ T (NCD8) cells, cytotoxic T cells (CTL), Treg cells, T follicular helper (Tfh) cells, Th17 cells, Th1 cells, and proliferative T (Pro-T) cells (Fig. 2a–c). During EAU, the proportion of Th17 and Th1 cells, two effector T cell subsets in EAU^20^, increased in the first 2 weeks, and then decreased (Fig. 2d). Pro-T cells also showed a similar tendency, but peaked on day 7 (Fig. 2d). Next, we evaluated the expression of *Pim1* in each T cell subset. *Pim1* was noticeably upregulated in Th17 cells, Th1 cells and Treg cells (Fig. 2e). Among them, Th17 cells and Treg cells are the core regulators of the pathogenesis of uveitis^21^, suggesting that PIM1 may involve in Th17/Treg cell imbalance during uveitis.

To investigate the dynamic changes in the T cell subset transcriptome, we conducted GO analysis on the DEGs of each T cell subset. On day 7, the main enriched pathways in most T cell subsets were annotated as “cellular responses to stress and stimuli” (Supplementary Fig. 3a). In EAU, several pathways were uniquely enriched on day 7 compared to the control group, such as the TNF signaling pathway in CTLs, naïve CD4^+^ T cells, and Tfh cells; the leukocyte differentiation pathway in naïve CD4^+^ T cells; and the IL-17 signaling pathway in naïve CD4^+^ T cells and Th17 cells (Supplementary Fig. 3b). On day 14, the above uniquely enriched pathways in EAU remained evident and pathways annotated as “cellular responses to stress and stimuli” were enriched in all T cell subsets compared to the control group (Fig. 2f, g). On day 21, the electron transport chain and ATP metabolic process pathways were widely enriched in T cell subsets in EAU (Supplementary Fig. 3c, d).

Collectively, the evident upregulation of PIM1 expression in Th17 cells and Treg cells indicated the potential involvement of PIM1 in EAU pathogenesis. Additionally, GO analysis of T cell subsets showed enriched pathways in different T cell subsets and time points.

### Inhibiting PIM1 alleviated EAU

**Fig. 3:**
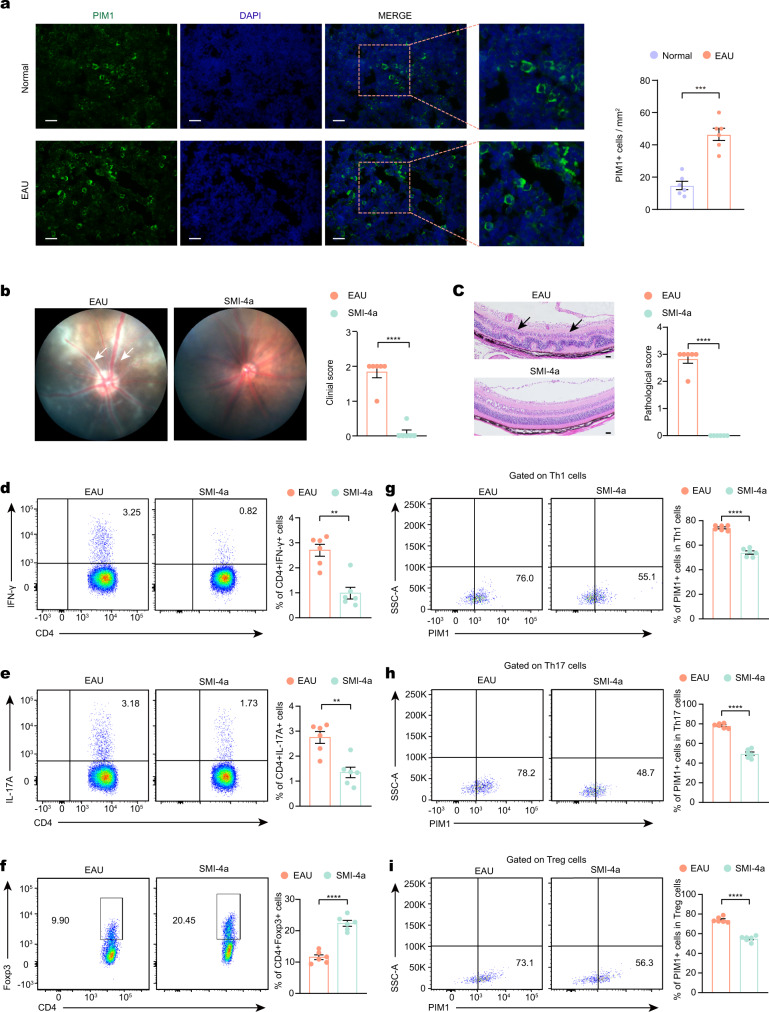
Inhibiting PIM1 alleviated EAU. **a** Immunostaining of cross-sections of CDLNs from day 0 group and day 14 EAU group shows PIM1 (green) and nuclei (4′,6-diamidino-2-phenylindole [DAPI]; blue). Each group contains six mice. *P*(EAU-Normal) = 0.0003. Data represented as mean ± SEM. Significance was determined using unpaired two-tailed student’s *t* test. ***P < 0.001. Scale bars, 20 mm. **b** Representative fundus images and clinical scores of eyes from the vehicle group and SMI-4a group after immunization at day 14. White arrowheads indicate inflammatory exudation and vascular deformation. Each group contains six mice. *P*(EAU-SMI-4a) = 2.8E-06. Data represented as mean ± SEM. Significance was determined using unpaired two-tailed student’s *t* test. ****P < 0.0001. **c** Representative histopathological images (hematoxylin and eosin staining) and pathological scores of eyes from the vehicle group and SMI-4a group after immunization at day 14. Black arrowheads indicate infiltration of inflammatory cells and retinal folding. Each group contains six mice. *P*(EAU-SMI-4a) = 1.0E-08. Data expressed as mean ± SEM. Significance was determined using unpaired two-tailed student’s *t* test. ****P < 0.0001. Scale bars, 20 mm. **d**–**f** Proportions of Th1 cells (**d**), Th17 cells (**e**) and regulatory T cells (Treg) (**f**) were measured by flow cytometry after immunization at day 14. Each group contains six mice. *P*(Th1) = 0.0005, *P*(Th7) = 0.0014, *P*(Treg) = 3.8E-06. Data expressed as mean ± SEM. Significance was determined using unpaired two-tailed student’s *t* test. *P < 0.05, **P < 0.01, ****P < 0.0001. **g**–**i** Proportions of PIM^+^ cells in Th1 cells (**g**), Th17 cells (**h**) and regulatory T cells (Treg) (**i**) were measured by flow cytometry after immunization at day 14. Each group contains six mice. *P*(Th1) = 1.8E-07, *P*(Th7) = 7.4E-08, *P*(Treg) = 3.5E-07. Data expressed as mean ± SEM. Significance was determined using unpaired two-tailed student’s *t* test. ****P < 0.0001.

In the above analysis, we identified the upregulation of PIM1 in EAU at transcriptional level. PIM1 is a member of the PIM kinase family, which is actively involved in cell proliferation, survival, and apoptosis in many diseases^22,23^. To evaluate the involvement of PIM1 in EAU, we conducted immunostaining of CDLNs for PIM1 and verified its enhanced expression in EAU at the protein level (Fig. 3a). Next, SMI-4a, a PIM1 inhibitor^24^, was administered to EAU mice. EAU symptoms were significantly reduced in mice treated with the inhibitor, as determined by fundus examination and histopathological analysis (Fig. 3b, c; Supplementary Fig. 3e). Moreover, flow cytometry of CDLN cells showed that SMI-4a treatment reduced the proportion of Th1 and Th17 cells and enhanced the proportion of Treg cells compared to the vehicle-treated group (Fig. 3d–f). Reduced proportion of PIM1^+^ cells among Th1 cells, Th17 cells, Treg cells, and Tfh cells after SMI-4a treatment was also verified by flow cytometry (Fig. 3g–i; Supplementary Fig. 3f, g). Furthermore, we treated EAU mice with another PIM1 highly selective inhibitor, quercetagetin^25^. Quercetagetin also effectively alleviated EAU symptoms (Supplementary Fig. 3h, i). These results indicated that PIM1 may exacerbate EAU via regulation of CD4^+^ T cell subsets.

### PIM1 regulates Th17/Treg cell balance

**Fig. 4:**
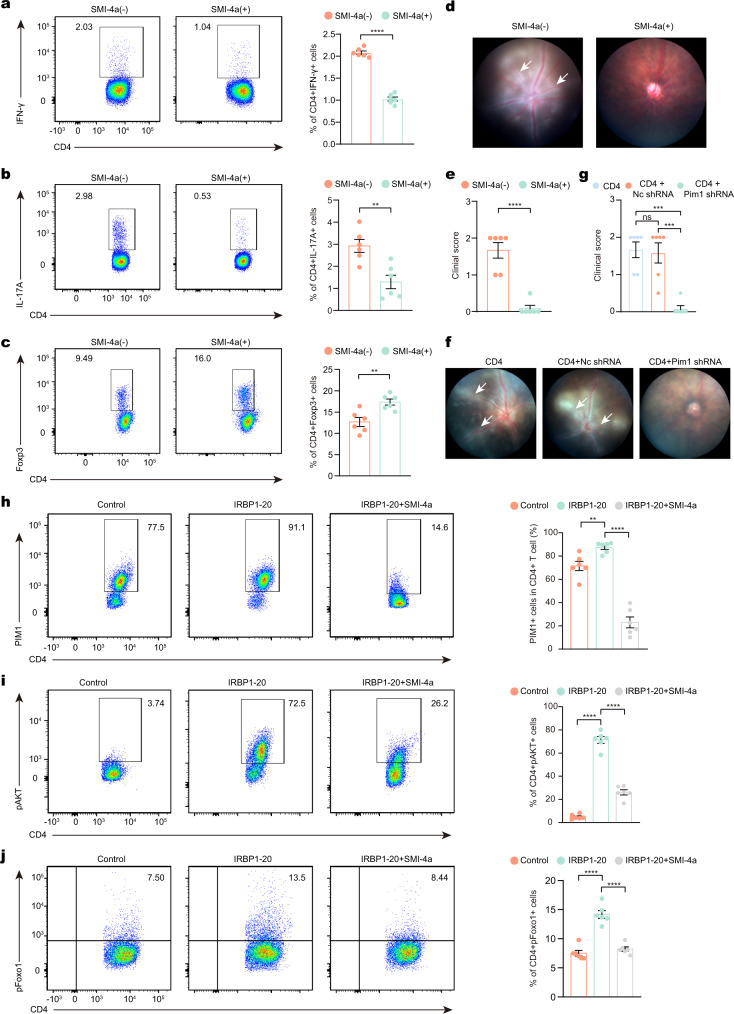
Pim1 regulated the balance of Th17/Treg cell. **a**–**c** CDLN cells from EAU mice were cultured with IRBP1-20 or IRBP1-20 plus SMI-4a (20 μM). Flow cytometry was performed to show the proportion of Th1 cells (**a**), Th17 cells (**b**) and Treg cells (**c**). Each group contains six mice. *P*(Th1) = 9.3E-09, *P*(Th7) = 0.0030, *P*(Treg) = 0.0040. Data expressed as mean ± SEM. Significance was determined using unpaired two-tailed student’s *t* test. **P < 0.01. **d**, **e** Representative fundus images (**d**) and clinical score (**e**) after induction of CD4^+^ T cells cultured with IRBP1-20 or IRBP1-20 plus SMI-4a at day 14. White arrowheads indicate inflammatory exudation and vascular deformation. Each group contains six mice. *P*(EAU-SMI-4a) = 3.8E-05. Data expressed as mean ± SEM. Significance was determined using unpaired two-tailed student’s *t* test. ****P < 0.0001. **f**, **g** Representative fundus images (**f**) and clinical score (**g**) after induction of Pim1 shRNA treated-CD4^+^ T cells cultured with IRBP1-20 at day 14. Each group contains six mice. *P*(CD4-CD4 + Nc shRNA) = 0.9552, *P*(CD4-CD4 + pim1 shRNA) = 0.0002, *P*(CD4 + Nc shRNA- CD4 + pim1 shRNA) = 0.0003. Data expressed as mean ± SEM. Significance was determined using one-way ANOVA. ns no significant differences, ***P < 0.001. **h**–**j** CD4 T cells from EAU group cultured with IRBP1-20 alone or with IRBP1-20 plus with SMI-4a for 72 h. Flow cytometry showed the proportion of PIM1^+^ cells (**h**), pAKT^+^ cells (**i**), and pFOXO1^+^ cells (**j**) in total CD4^+^-gated T cells. Data represented as mean ± SEM from six independent experiments. *P*(PIM1^+^ cells,Control-IRBP1-20) = 0.0208, *P*(PIM1^+^ cells, IRBP1-20-IRBP1-20+SMI-4a) = 8.2E-09, *P*(pAKT^+^ cells,Control-IRBP1-20) = 3.8E-12, *P*(pAKT^+^ cells, IRBP1-20-IRBP1-20+SMI-4a) = 8.6E-10, *P*(pFOXO1^+^ cells cells,Control-IRBP1-20) = 4.4E-07, *P*(pFOXO1^+^ cells, IRBP1-20-IRBP1-20+SMI-4a) = 1.8E-06. Significance was determined using two-way ANOVA. **P < 0.01, ****P < 0.0001.

To further explore the function of PIM1 in EAU, in vitro experiments were conducted using SMI-4a. SMI-4a at 20 μm did not affect cell viability but effectively repressed the proliferation of CD4^+^ T cells (Supplementary Fig. 4a–d). Therefore, we chose this dose for the following experiments. CDLN cells were isolated from mice in EAU groups and cultured with IRBP_1–20_ for 72 h in the presence or absence of SMI-4a. It was noted that the proportion of Th1 and Th17 cells decreased in the presence of SMI-4a, whereas that of Treg cells increased (Fig. 4a–c). Meanwhile, we transferred SMI-4a-treated or non-treated CD4^+^ T cells into mice and observed that the pathogenicity of CD4^+^ T cells was dampened by SMI-4a treatment (Fig. 4d, e). Similar experiments were conducted using *Pim1*-targeting short hairpin RNA (*Pim1* shRNA). *Pim1* shRNA induced similar changes in the proportion of CD4^+^ T cell subsets (Supplementary Fig. 5a–d) and prevented CD4^+^ T cells to induce EAU in adoptive transfer experiments (Fig. 4f, g). These results indicated that PIM1 is indispensable for the pathogenicity of CD4^+^ T cells in EAU.

The balance between Th17 cells and Treg cells is the core regulator of uveitis pathogenesis^20,21,26^. Thus, we further explored the regulatory function of PIM1 on Th17/Treg cell balance. Protein kinase B (AKT) is regulated by PIM1^27^. In addition, the AKT/Forkhead box protein O1 (FOXO1) pathway has been shown to regulate the balance between Th17 cells and Treg cells, and involve in several diseases, such as multiple sclerosis^28,29^. In the AKT/FOXO1 pathway, when FoxO1 locates in the nucleus, it enhances Treg cell function via maintaining the expression of FOXP3, but represses the differentiation of Th17 cells by inhibiting the expression of RORγ^29,30^. Phosphorylated AKT can directly phosphorylate FOXO1, which induces FOXO1 to leave the nucleus, relocate in the cytosol, and lose its regulatory function in Th17 cells and Treg cells^29^. To explore the activity of the PIM1/AKT/FOXO1 pathway in EAU pathogenesis, we isolated CDLN cells from EAU mice, treated these cells with IRBP_1–20_ or IRBP_1–20_ plus SMI-4a, and assessed the levels of PIM1, pAKT, and pFOXO1. Flow cytometry demonstrated that IRBP_1–20_ treatment increases the proportion of PIM1^+^ cell and stimulated the phosphorylation of AKT and FoxO1 in total CD4^+^ T cells. These IRBP_1–20_-associated effects were reversed by SMI-4a treatment (Fig. 4h–j). These results indicated that an abnormal PIM1/AKT/FOXO1 pathway in EAU may contribute to Th17 /Treg cell imbalance in uveitis, which may be ameliorated by PIM1inhibiting.

Within CD4^+^ T cell subsets, Th17 cells are known to be the critical pathogenic cell type in EAU^10^. Transfer of autoreactive Th17 cells can induce EAU development^10^. Therefore, we isolated CD4^+^ CCR6^+^ CXCR3^−^ T cells (Th17 cells)^31^ from EAU mice. *Pim1* shRNA treatment abrogated the capacity of Th17 cells to transfer the disease to naïve mice (Supplementary Fig. 5e), which further indicated that PIM1 is essential for the pathogenicity of Th17 cells, and therefore the disease.

In summary, by inhibiting PIM1, we identified that PIM1 regulates the balance between Th17 cells and Treg cells, which may result from its downstream AKT/FOXO1 signaling. Additionally, PIM1 is indispensable for the pathogenicity of Th17 cells in EAU.

### Depleting B cells ameliorated EAU

**Fig. 5:**
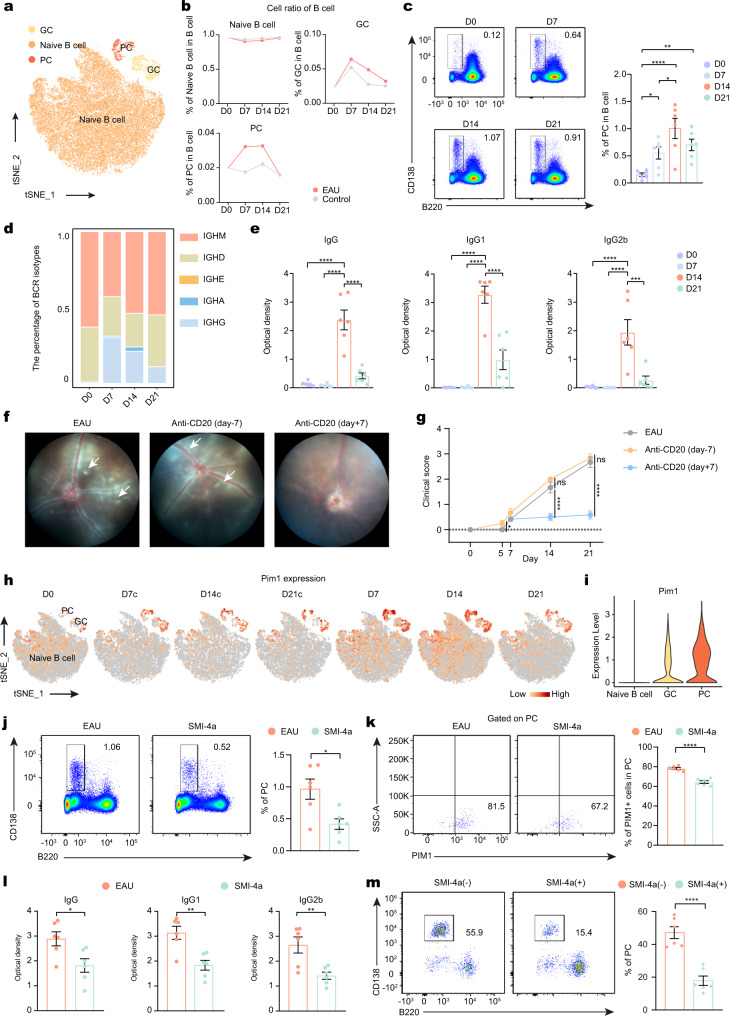
Depleting B cells ameliorated EAU and PIM1 regulated PCs differentiation. **a** t-SNE plots of B cell subsets from all mice groups. **b** Proportion of B cell subsets. **c** Frequency of PCs. Each group contains six mice. *P*(D0-D7) = 0.0316, *P*(D0-D14) = 7.7E-05, *P*(D0-D21) = 0.0047, *P*(D7-D14) = 0.0157. Data represented as mean ± SEM. Significance was determined using one-way ANOVA. *P < 0.05, **P < 0.01, ****P < 0.0001. **d** Percentage of BCR isotype. **e** Concentration of IRBP1-20-specific antibodies. Each group contains six mice. *P*(IgG, D0-D14) = 1.8E-07, *P*(IgG, D7-D14) = 1.2E-07, *P*(IgG,D14-D21) = 1.4E-06, *P*(IgG1, D0-D14) = 1.6E-08, *P*(IgG1, D7-D14) = 1.7E-08, *P*(IgG1,D14-D21) = 4.1E-06, *P*(IgG2b, D0-D14) = 6.2E-05, *P*(IgG2b, D7-D14) = 5.1E-05, *P*(IgG2b,D14-D21) = 0.0003. Data represented as mean ± SEM. Significance was determined using one-way ANOVA. ***P < 0.001, ****P < 0.0001. **f** Administration of anti-CD20 antibodies to deplete the B cells before (7 days before immunization, day −7) and after EAU (7 days after immunization, day +7). Representative fundus images of eyes after immunization at day 14. White arrowheads indicate inflammatory exudation. **g** Clinical scores after immunization at day 14. Each group contains six mice. *P*(D5, EAU- Anti-CD20 (day-7)) = 0.0493, *P*(D14, EAU- Anti-CD20 (day-7)) = 0.2557, *P*(D14, EAU- Anti-CD20 (day + 7)) = 6.0E-06, *P*(D21, EAU- Anti-CD20 (day-7)) = 0.7900, *P*(D21, EAU- Anti-CD20 (day + 7)) = 1.7E-06. Data represented as mean ± SEM. Significance was determined using one-way ANOVA. ns, no significant differences, *P < 0.05, ****P < 0.0001. **h** t-SNE plots of Pim1 by B cell subsets. **i** Violin plots of Pim1 in B cell subsets from all mice groups. **j** Proportions of PCs after immunization at day 14. Each group contains six mice. *P*(EAU-SMI-4a) = 0.0114. Data expressed as mean ± SEM. Significance was determined using unpaired two-tailed student’s *t* test. *P < 0.05. **k** Proportions of PIM1^+^ cells in PCs after immunization at day 14. Each group contains six mice. *P*(EAU-SMI-4a) = 1.7E-06. Data expressed as mean ± SEM. Significance was determined using unpaired two-tailed student’s *t* test. ****P < 0.0001. **l** Concentration of IRBP1-20-specific antibodies. Each group contains six mice. *P*(IgG) = 0.0207, *P*(IgG1) = 0.0027, *P*(IgG2b) = 0.0058. Data expressed as mean ± SEM. Significance was determined using unpaired two-tailed student’s *t* test. *P < 0.05, **P < 0.01. **m** Proportion of PCs. Data represented as mean ± SEM from six independent experiments. *P*(SMI-4a (-)-SMI-4a (+)) = 8.4E-05. Significance was determined using unpaired two-tailed student’s *t* test. ****P < 0.0001.

Although the function of B cells in EAU is less certain than that of T cells, increasing evidence supports their pathogenic functions in other autoimmune diseases, such as multiple sclerosis^9,32,33^. Thus, we re-clustered B cells and identified three B cell subtypes, including germinal center B cells (GC), naïve B cells, and PCs (Fig. 5a; Supplementary Fig. 6a, b). The proportion of PCs showed a trend from increasing to decreasing which peaked on days 14 (Fig. 5b, c).

To investigate the dynamic changes in the transcriptome of B cell subsets in EAU, we conducted GO analysis on the DEGs of each B cell subset. B cell subsets showed various pathways that were exclusively enriched in EAU at different time points. For example, the protein folding and export pathways were enriched in plasma cells on day 7; the leukocyte differentiation and adaptive immune response pathways were enriched in naïve B cells on day 14; and the lymphocyte activation and B cell differentiation pathways in naïve B cells and ATP metabolic process pathway in GCs were enriched on day 21 (Supplementary Fig. 6c).

Subsequently, we explored antibody production, a critical process in many autoimmune diseases, in EAU. To obtain isotype information of immunoglobulin, B-cell receptor (BCR) sequences were extracted from B cell transcriptomes and analyzed. As shown in Fig. 5d, the percentage of IGHG and IGHA was increased in EAU, which implicated isotype switching of immunoglobulins from IgM/IgD to IgG/IgA resulted from B-cell activation in EAU. ELISA was performed to evaluate serum levels of anti-IRBP_1–20_ IgG antibodies and their isotypes (IgG1, IgG2a, IgG2b, and IgG3, but not IgG2c) in the day 0 group and EAU groups at different time points (Fig. 5e; Supplementary Fig. 6d). The serum levels of anti-IRBP_1–20_ total IgG and its isotypes IgG1 and IgG2b increased in EAU groups after day 7, reaching a maximum level on day 14, and then decreased (Fig. 5e). The serum levels of IgG2a and IgG3 did not differ significantly at each time point (Supplementary Fig. 6d).

In EAE, an animal model showing many similarities in the pathogenic mechanisms with EAU^17,34^, B cells reportedly have dual functions depending on the disease stages: anti-CD20 antibody–mediated B cell depletion before EAE induction exacerbates disease symptoms, but B cell depletion during EAE disease progression suppresses disease symptoms^35^. To explore the function of B cells in EAU, we administrated anti-CD20 antibodies to deplete B cells before (7 days before immunization, day −7) and after EAU development (7 days after immunization, day +7) (Fig. 5f, g). Flow cytometry validated the dramatically decreased B cell proportion in the two anti-CD20 antibody treatment groups (Supplementary Fig. 6e). Depleting B cells before the induction of EAU accelerated disease initiation, with EAU developing on day 5 (Fig. 5g). However, depleting B cells after EAU development ameliorated EAU symptoms (Fig. 5g). These results indicated that B cells mainly have protective effect in the initiation stage of EAU and exert a pathogenic function during EAU.

Considering the enhanced anti-IRBP_1–20_ antibodies in EAU, we speculated that PCs might also contribute to EAU pathogenesis. PCs are thought to be particularly sensitive to bortezomib (BZ), a proteasome inhibitor, due to their high rate of antibody production^36^. In the current study, BZ was administered to EAU mice twice a week for 2 weeks. After treatment with BZ, EAU symptoms were ameliorated, as shown by lower clinical and pathological scores (Supplementary Fig. 7a–c). Further, the percentage of PCs within B cells and serum levels of anti-IRBP_1–20_ IgG and its isotypes (IgG1 and IgG2b) in EAU mice were reduced after BZ treatment (Supplementary Fig. 7d, e). BZ also inhibited PC differentiation in vitro experiments (Supplementary Fig. 7f). We also evaluated the influence of BZ administration on the PIM1/AKT/FOXO1 pathway in total CD4^+^ T cells. BZ did not influence the expression of PIM1 and phosphorylation of AKT, but inhibited the phosphorylation of FOXO1 (Supplementary Fig. 7g–i). These results indicated that PC depletion by BZ might contribute to the amelioration of EAU.

### PIM1 regulated PC differentiation

We then evaluated the expression and function of PIM1 in B cell subsets. *Pim1* was mainly expressed by PCs in the scRNA-seq data (Fig. 5h, i). SMI-4a treatment reduced the proportion of PCs and serum levels of anti-IRBP_1–20_ IgG and its isotypes (IgG1 and IgG2b) in EAU mice, as shown by flow cytometry and ELISA (Fig. 5j, l). The proportion of PIM1^+^ cells in PCs and GCs was reduced by SMI-4a (Fig. 5k; Supplementary Fig. 8a, b). We also investigated the function of PIM1 in the differentiation of PCs in vitro. Sorted B cells were cultured for 5 days with CD40L, IgM F(ab′)2, IL-4, and IL-5, with or without SMI-4a. SMI-4a treatment decreased the proportion of PCs (B220^+^ CD138^+^) (Fig. 5m). Similarly, *Pim1* shRNA inhibited PC differentiation (Supplementary Fig. 8c, d). We then evaluated the effects of SMI-4a on the AKT/FOXO1 pathway in plasma cells. SMI-4a reduced the phosphorylation of AKT, but did not significantly influence the expression of pFOXO1 in plasma cells (Supplementary Fig. 8e, f). These results indicated that PIM1 may engage in PC differentiation and this effect may also contribute to EAU pathogenesis.

### Validating the function of PIM1 in human Vogt-Koyanagi-Harada disease

VKH, one of the most common types of human uveitis in Asia, is characterized by a type of bilateral uveitis frequently associated with neurological (meningeal), auditory, and integumentary manifestations^37^. To investigate whether the upregulation of PIM1 expression is conserved in human uveitis, we generated scRNA-seq data from peripheral blood mononuclear cells (PBMC) obtained from six patients with VKH and six healthy controls (HC). Five immune cell types were recognized based on classical lineage markers (Supplementary Fig. 9a–d). We then conducted DEG and GO analyses to identify the transcriptional changes in human VKH (Supplementary Fig. 9e, f). In total PBMCs, DEGs related to cell activation (*CD69*), IL-17 signaling (*JUNB*), inflammation (*S100A8* and *S100A9*), and *PIM1* were upregulated in patients with VKH compared to HCs (Supplementary Fig. 9e). Correspondingly, DEGS in patients with VKH were enriched in pathways related to chronic inflammatory response, IL-17 signaling, and T cell activation compared to HCs (Supplementary Fig. 9f). In T cells, *CD69*, *JUNB*, and *PIM1* were upregulated and the T cell activation pathway was enriched in patients with VKH (Supplementary Fig. 10a, b). Additionally, immunoglobulin-related genes (*IGHG1* and *IGHA1*) and pathway were upregulated in B cells of patients with VKH (Supplementary Fig. 10c, d). These transcriptional changes in patients with VKH were analogous to those observed in EAU mice.

**Fig. 6:**
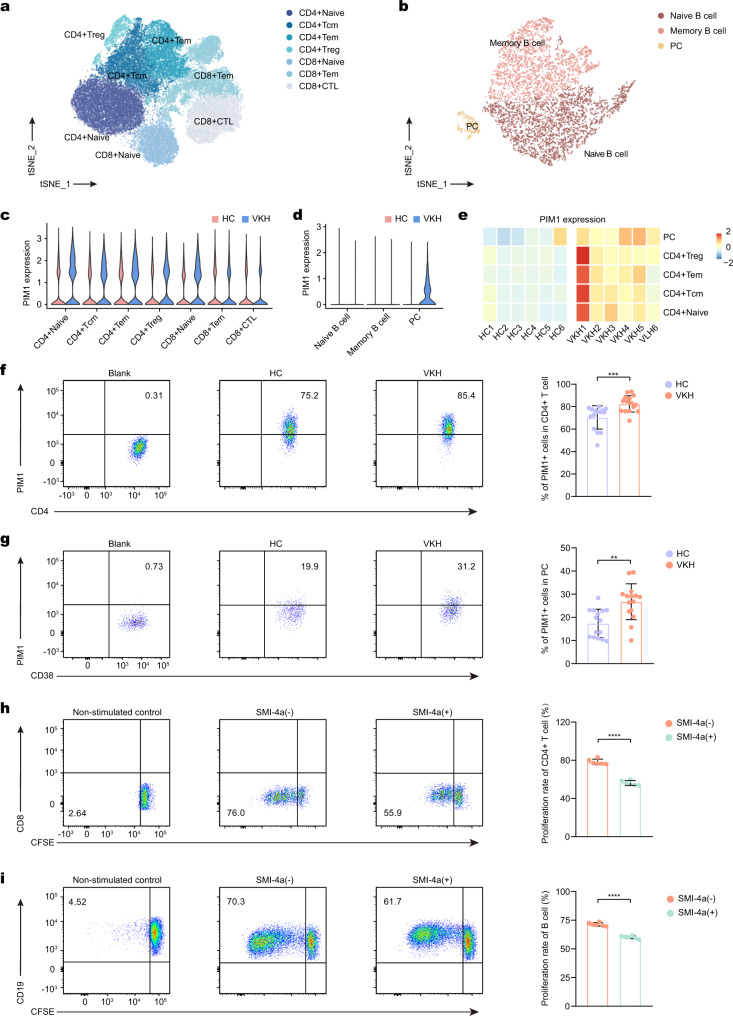
Upregulation of PIM1 in VKH patients. **a** t-SNE plots of T cell subsets from all healthy controls and VKH samples. **b** t-SNE plots of B cells subsets from all healthy controls and VKH samples. **c** Violin plots of PIM1 expression in T cell subsets of healthy controls and VKH patients. **d** Violin plots of PIM1 expression in B cell subsets of healthy controls and VKH patients. **e** Heatmap of the average expression of PIM1 by PCs and CD4 T cell subsets in healthy controls and VKH patients. **f**, **g** The percentage of PIM1^+^ cells in CD4 T cells (**f**) and PCs (**g**) measured by flow cytometry. Each group contains 15 samples. *P*(CD4 T cells, VKH-HC) = 0.0010, *P*(PCs, VKH-HC) = 0.0011. Data expressed as mean ± SEM. Significance was determined using unpaired two-tailed student’s *t* test. **P < 0.01, ***P < 0.001. **h**, **i** The proliferating rate of CD4 T cells (gated on CD3^+^CD8^-^ T cells) (**h**) and B cells (**i**) measured by flow cytometry. *P*(CD4 T cells, SMI-4a (−)-SMI-4a (+)) = 6.4E-08, *P*(PCs, SMI-4a (−)-SMI-4a (+)) = 7.1E-08. Data represented as mean ± SEM from six independent experiments. Significance was determined using unpaired two-tailed student’s *t* test. ****P < 0.0001.

Next, we re-clustered T cells and identified seven known T cell subsets in human PBMCs, including CD4^+^ naïve T (CD4^+^ naïve) cells, CD4^+^ central memory T (CD4^+^ Tcm) cells, CD4^+^ effector memory T (CD4^+^ Tem) cells, CD4^+^ Treg cells, CD8^+^ naïve T (CD8^+^ naïve) cells, CD8^+^ Tem cells, and CD8^+^ cytotoxic T cells (CD8^+^ CTL) (Fig. 6a; Supplementary Fig. 10e, f). B cells were also re-clustered into three B cell subsets incorporating naïve B cells, memory B cells, and plasma B cells (PC) (Fig. 6b; Supplementary Fig. 10g, h). All CD4^+^ T cell subsets and PCs showed upregulated *PIM1* expression in patients with VKH compared to HCs (Fig. 6c–e). At the protein level, CD4^+^ T cells and PCs exhibited increased PIM1 expression in patients with VKH (Fig. 6f, g; Supplementary Fig. 11a, b). SMI-4a at 20 μm did not influence cell viability but effectively repressed cell proliferation (Supplementary Fig. 11c–f). Therefore, we chose this dose for the following experiments. CD4^+^ T cells and B cells treated with SMI-4a showed decreased expansion compared to the untreated group (Fig. 6h, i). Similarly, *PIM1* shRNA inhibited the proliferation of CD4^+^ T cells and B cells (Supplementary Fig. 12a–f). Additionally, we found that the addition of SMI-4a after *PIM1* shRNA treatment only slightly further inhibited the proliferation of CD4^+^ T cells (Supplementary Fig. 12b, c). Thus, the upregulation of PIM1 expression and therapeutic function of PIM1 inhibitors may extend to VKH.

To explore whether the upregulation of PIM1 was unique to uveitis, we downloaded the scRNA-seq data of patients with MS from a previous study^38^ and analyzed *PIM1* expression in this dataset (Supplementary Fig. 13a–f). *PIM1* expression in PBMCs from patients with MS showed similar alterations to that in patients with VKH disease. *PIM1* expression was upregulated in nearly all T cell subsets (Supplementary Fig. 13g, i). *PIM1* expression was also upregulated in PCs (Supplementary Fig. 13h, i). Therefore, increased PIM1 expression and potential therapeutic value of targeting PIM1 may not be limited to uveitis but may also be applied to other CNS autoimmune diseases, such as MS.

## Discussion

Uveitis is one of the major causes of blindness^2^. It pertains to CNS autoimmune diseases and is characterized by aberrant infiltration of large numbers of leukocytes into the uvea, vitreous, retina, or sclera^1,4^. CDLNs can be used to measure immune drainage from the CNS and detect changes earlier than those in the retina^39^. Here, single-cell-based transcriptional profiling of CDLNs from EAU mice showed dynamic and specific disease-associated alterations in cell type composition and transcriptional regulation. Our results showed an increase in the proportion of Th17 cells and PCs during EAU, reaching a peak on day 14. These cells showed high PIM1 expression. Subsequent studies demonstrated that PIM1 was involved in EAU pathogenesis. We identified the PIM1-regulated AKT/FOXO1 pathway as being critical for the balance of Th17 cells and Treg cells. Inhibiting PIM1 reduced the proportion of Th17 cells and increased the proportion of Treg cells among CD4^+^ T cells in EAU in vivo and in vitro. CD4^+^ T cells and Th17 cells failed to induce EAU when their expression of PIM1 was repressed. Moreover, our study validated that B cells contribute to EAU pathogenesis. Additionally, inhibiting PIM1 in B cells reduced PC differentiation. Importantly, the upregulation of PIM1 and its regulatory function were conserved in a human uveitis, VKH. Therefore, PIM1 may act as a potential therapeutic target for uveitis.

Conventional application of bulk RNA-seq has improved our understanding of the pathogenic mechanisms of numerous diseases, but only provides a virtual average of diverse constituent cells^40^. ScRNA-seq enables deep analysis of the gene expression profiles of different cell subsets within a complex population mix^40^. ScRNA-seq of LNs has been used to demonstrate the pathogenic mechanisms of many diseases, including cancer, infections, and atherosclerosis^41–43^. In this study, we explored EAU-specific transcriptional changes by utilizing pertussis toxin and complete Freund’s adjuvant-treated mice as control groups to exclude simple immunization- or manipulation-related effects. As shown by the DEG and GO analysis of the global CDLN cells, inflammation was present in control groups, which was supported by enrichment of the lymphocyte activation, immune response, and immunoglobulin production pathways. On the other hand, the IL-17 signaling pathway, which was uniquely enriched in EAU, represents an EAU-specific change. The importance of CD4^+^ T cells during uveitis and other CNS autoimmune diseases has long been recognized^44^. CD4^+^ effector T cells, especially IL-17-secreting Th17 cells, are critical in autoimmune disorders, whereas Treg cells mediate the maintenance and tolerance of autoimmunity^45^. The increased proportion of Th17 cells, as well as the enriched IL-17 signaling pathway in EAU suggested by our scRNA-seq data, indicating their involvement in EAU pathogenesis. Myeloid cells are important pro-inflammatory cells that produce inflammatory cytokines and mediate tissue injury during autoimmune diseases^46,47^. They also act as critical antigen-presenting cells^46^. In our scRNA-seq analysis, we observed amplified inflammatory gene signatures of several myeloid cells compared to control groups, as well as expanded antigen processing and presentation in DCs. Thus, adaptive and innate immune cells cooperate to promote EAU progression. In addition, our study provides abundant transcriptional information during EAU progression, which may serve as a source for other time-associated studies on uveitis.

As crucial components of the adaptive immune system, B cells have a wide range of functions, including antibody secretion, antigen presentation, and cytokine production^48^. Studies have demonstrated the pathological function of B cells in various autoimmune diseases, such as rheumatoid arthritis^49^ and systemic lupus erythematosus^50^. Although the specific function of B cells in uveitis is not fully understood, their function in multiple sclerosis, another CNS autoimmune disease, has garnered enormous interest because of the success of anti-CD20 therapy^51^. CD20 is a B cell-specific molecule that is first expressed on the cell surface during the pre- B cell to immature B cell transition and is lost upon plasma cell differentiation^52^. A previous study reported that anti-CD20 antibody-mediated B cell depletion before the induction of EAE substantially exacerbated disease symptoms, whereas B cell depletion after EAE development ameliorated disease symptom^35^. These results demonstrated that except for pathogenic B cell subsets, regulatory B cells also exist, and this B cell subset dominates the induction phase of EAE. Similarly, regulatory PCs were also identified^53^. These studies provided a methodological reference for exploring the function of B cells or PCs in autoimmune diseases. In our study, similar experiment strategy was taken and we observed that depletion of B cells after EAU development ameliorated disease symptoms while treatment with anti-CD20 antibody before EAU induction hastened disease initiation, a pattern highly consistent with that observed in EAE. These results indicated that regulatory B cells dominated in the initiation stage of EAU, while B cells mainly have a pathogenic function during EAU. Some clinical evidence supports the therapeutic efficacy of rituximab, an anti-CD20 antibody, against refractory chronic recurrent uveitis associated with VKH^32^. This clinical evidence was consistent with the pathogenic function of B cells during EAU. Our study further attempted to explore the function of PCs during EAU. Since specially depleting PCs without influencing other cell types after EAU development is challenging, we used BZ, a proteasome inhibitor, to repress PCs during EAU. The rationale for this was that PCs were thought to be particularly sensitive to bortezomib because of their high rate of antibody synthesis^54^. Previous studies have reported that BZ can deplete plasma cells and effectively treat multiple myeloma (plasma cell malignancy) and systemic lupus erythematosus^36,55,56^. Here, BZ effectively depleted PCs in EAU, lowered circulating autoantibody levels, and ameliorated the severity of EAU, suggesting that PC depletion by BZ might contribute to the amelioration of EAU symptoms. Taken together, our study findings confirm the pathogenicity of B cells during EAU and suggest the potential involvement of PCs in EAU pathogenesis.

The function of PIM1 in cancer has been widely investigated and PIM1 is known to be an oncogenic protein^19^. Apart from its function in promoting cancer cell proliferation and inhibiting apoptosis, PIM1 has been reported to regulate cancer immunology^19^. Many factors, such as inflammation, hypoxia, and growth factors, are related to the upregulation of PIM1^19^. However, its function and underlying molecular regulation in uveitis is poorly understood. Our study showed the upregulation of PIM1 in CDLN cells during EAU, and PIM1 inhibiting ameliorated EAU symptoms. Additionally, the upregulation of inflammation-related genes (*S100a8* and *S100a9*) and enriched GO terms related to IFN-γ, inflammation, and TNF pathways, which may contribute to the upregulation of PIM1 during EAU, were also shown in our scRNA-seq data. We also observed a higher expression of *Pim1* in Th1 cells, Th17 cells, Treg cells, and PCs than in Tfh cells and GC B cells during EAU. After treatment with SMI-4a, the proportion of PIM1^+^ cells also decreased more in Th1 cells, Th17 cells, Treg cells, and PCs than in Tfh cells and GC B cells. These results indicated that PIM1 may be preferentially expressed in Th17 cells, Th1 cells, Treg cells, and PCs during EAU, and these cells may be affected more by PIM1 inhibitors because of their higher expression of PIM1.

Among the above cells, Th17 cells and Treg cells are the core CD4^+^ T cell subsets closely related to EAU pathogenesis^21^. High expression of PIM1 in Th17 cells and Treg cells indicated the regulatory potential of PIM1 in these two CD4^+^ T cell subsets and, therefore, in EAU. Indeed, inhibition of PIM1 decreased the proportion of Th17 cells but increased the proportion of Treg cells in vivo and in vitro. In addition, transfer of PIM1 inhibitor/*Pim1* shRNA-treated CD4^+^ T cells failed to induce EAU. These results suggest that PIM1 may regulate the Th17/Treg cell balance, thus contribute to EAU pathogenesis. Therefore, we further explored the downstream AKT/FOXO1 signaling pathway of PIM1. Ser/Thr kinase AKT is regulated by PIM1^27^ and, once activated, AKT phosphorylates its downstream molecule FOXO1. FOXO1 can directly inhibits Th17 cell differentiation and is essential for the suppressive function and generation of Treg cells^29^. Phosphorylation of FOXO1 causes it to re-localize to the cytosol and lose its function^29^. In our experiments, CD4^+^ T cells from EAU mice exhibited increased PIM1 expression as well as phosphorylation of AKT and FOXO1 in response to autoantigen challenge, whereas inhibition of PIM1 reversed these changes. Thus, aberrant PIM1/AKT/FOXO1 signaling may mediate Th17/Treg cell imbalance during EAU. To further investigate the cellular specificity of PIM1 in EAU, we conducted adoptive transfer experiments with Th17 cells. Notably, our adoptive transfer experiments showed that transfer of Th17 cells treated with *Pim1* shRNA failed to induce EAU. This result indicates that PIM1 is critical for the pathogenicity of Th17 cells.

The function of PIM1 is not limited to T cell subsets. Previous studies on the relationship between PIM1 and B cells have mainly focused on B cell lymphoma^57^, but the regulation of PC differentiation and B cell antibody production by PIM1 remain unclear. The results of our in vivo study indicated that inhibiting PIM1 reduced the proportion of PCs and their secretion of autoantibodies. Our in vitro study further confirmed that PIM1 is critical for PC differentiation. Combined with the function of B cells and PCs in EAU as discussed above, PIM1 may also engage in EAU pathogenesis via regulation of B cells and PCs.

In addition to CD4^+^ T and B cells, PIM1 functions in other immune cells^24,58,59^. These studies suggest the potential promoting effects of PIM1 on CD8^+^ T and inflammatory cells in uveitis. In addition to immune cells, PIM1 also regulates the function of blood endothelial cells^60^. Loss of PIM1 expression has been shown to enhance endothelial barrier integrity^60^, a function that regulates inflammation by controlling leukocyte passage through the wall of blood vessels. Thus, PIM1 expressed in endothelial cells may participate in EAU pathogenesis by regulating inflammatory cell recruitment. Although PIM1 is also expressed in other ocular cells, such as retinal ganglion cells, its functions are mainly associated with early eye development^61^ and repair of optic nerve damage^62^ and no involvement in EAU symptoms has been reported. Therefore, the effects of SMI-4a are likely both immunological and ocular in EAU, although the immunological impacts are the primary, based on our current results. Furthermore, according to the critical function of Th17 cells and the results of adaptive transfer experiments, PIM1 may exert its pathogenic effects predominantly via Th17 cells, although other cell types may also contribute to the pathogenic effect of PIM1. Importantly, upregulation of PIM1 expression was observed in patients with VKH at both the transcriptional and protein levels, and PIM1 inhibitor suppressed the expansion of CD4^+^ T cells and B cells, supporting PIM1 as a promising therapeutic target in VKH.

In summary, this study provides a comprehensive and dynamic single-cell atlas of CDLN cells during uveitis in multiple facets of the immune system, including cellular proportions, DEGs, and enriched pathways. Significantly, we identified PIM1 as an important regulator of Th17/Treg cell balance, Th17 cell pathogenicity, and PC differentiation during EAU. PIM1/AKT/FOXO1 signaling may be a potential mechanism of EAU pathogenesis, mediating the imbalance between Th17 cells and Treg cells, and we suggest that PIM1 inhibition may ameliorate EAU. These findings may extend to human uveitis, VKH, and other CNS autoimmune diseases.

## Methods

### Mice

Wild-type female C57BL/6 J mice (6–8 weeks old, 18–25 g) were purchased from the Medical Lab Animal Center (Guangzhou, China). All mice were housed in specific pathogen-free environment at 21 ± 1 °C and 60 ± 5% humidity, with a 12 h light/dark cycle. All experiments were conducted in accordance with the policies for animal health and usage. Animal experiments in our study were allowed by the Institutional Animal Care Committee (Zhongshan Ophthalmic Center, Sun Yat-Sen University).

### Human donors

Patients with VKH disease were enrolled at Zhongshan Ophthalmic Center, Sun Yat-sen University, Guangzhou, China. VKH was diagnosed based on disease manifestations and the results of standard coherent optical tomography and indocyanine green fluorescein angiography, according to the Revised Diagnostic Criteria (RDC) of VKH disease^63^. Written informed consent was obtained from all human donors. Blood samples were collected from six donors diagnosed with VKH and six healthy controls for scRNA-seq. Subsequently, in order to verify the function of PIM1 with flow cytometry, we recruited another fifteen patients with VKH and fifteen healthy controls. All the patients had newly onset VKH and did not receive any therapy. Individuals with comorbidities, including diabetes, hypertension, cancer, and other systemic diseases, were excluded. No remarkable sex or age differences were observed between patients with VKH and healthy controls (see Supplementary Table 3 for details). All protocols were reviewed and approved by the Medical Ethics Committee of the Guangzhou Zhongshan Ophthalmic Center (ID:2020KYPJ124).

### EAU model establishment

The mice were immunized with human IRBP1-20 (2 mg/mL; GiL Biochem, Shanghai, China) and complete Freund’s adjuvant (Difco, San Jose, CA, USA) containing 2.5 mg of *Mycobacterium tuberculosis* strain H37Ra (Difco) in a 1:1 volume ratio. In addition, 0.25 µg pertussis toxin in phosphate-buffered saline (PBS) was intraperitoneally injected on days 0 and 2 after immunization^64^. Funduscopic examination was performed to grade EAU mice with clinical scores from 0 to 3, according to the observable infiltration and vasculitis in the retina^64^. Clinical scores were evaluated in a blinded manner. See Supplementary Table 1 for detailed scoring criteria.

### Treatment protocols

The mice were intravenously injected twice weekly with BZ (*n* = 6; 0.75 mg/kg; Selleck Chemicals, Houston, TX, USA) or an equivalent volume of vehicle control (*n* = 6; PBS) for 2 weeks^55^.

Mice were orally treated with SMI‐4a (*n* = 6; 60 mg/kg; Selleck Chemicals) or vehicle control (*n* = 6; 0.1% DMSO/30% PEG-400/0.5% Tween80) once daily for 14 consecutive days after immunization^65^. For the in vitro experiments, 20 μM SMI-4a was used.

The mice were intravenously injected with anti-CD20 antibody (*n* = 6; 250 μg/mouse; Biolegend, San Diego, CA, USA) or an equivalent volume of vehicle control (*n* = 6; PBS) before (7 days before immunization, day −7) and after EAU development (7 days after immunization, day +7)^35^.

### CDLN cell isolation and treatment

CDLN cells were incubated with IRBP1-20 (20 μg/mL) with/without SMI-4a (20 μM) for 72 h. Harvested cells were used to further analysis.

### Adoptive transfer experiment

CDLN cells of EAU mice (day 14) stimulated with IRBP1-20 (20 μg/mL) with/without SMI-4a for 72 h. CD4^+^ T cells and CD4^+^ CCR6^+^ CXCR3^-^ cells (Th17 cells) were sorted, purified, and collected. Then, wild-type mice (*n* = 6) were injected with the above CD4^+^ T or Th17 cells (2 × 10^7^ living cells/mouse) through the tail vein. For experiment of *Pim1* shRNA, sorted CD4^+^ T or Th17 cells were incubated with lentivirus and polybrene (5 μg/mL) for 24 h, followed by Puromycin (5 μg/mL), and then transferred into wild-type mice (*n* = 6).

### Lentivirus transduction

PIM1 expression was knocked down using shRNA carried on a lentivirus vector (OBIO, Shanghai, China). The shRNA target sequences for PIM1 were 5′-GTCTCTTCAGAGTGTCAGCAC-3′ (mice)^66^ and 5′-GAAGGUGAGCUCGGGUUUC-3′ (human)^67^. The negative control shRNA sequence was 5′-TTCTCCGAACGTGTCACGT-3′. Cells were incubated with lentivirus and polybrene (5 μg/mL) for 24 h. Puromycin (5 μg/mL) was added to the culture medium to select transduced cells.

### Flow cytometry

After staining with live/dead dye, the harvested cells of mice were stained with surface markers. For mice samples, the cells were stained with Super Bright 702-anti-CD90.2 (Invitrogen, 67-0902-82, 0.2 μg/ml), Brilliant Violet 650-anti-F4/80 (Biolegend, 123149, 0.2 μg/ml), PE-anti-CD11c (BD Pharmingen, 557401, 0.2 μg/ml), eFluor 506-anti-CD45R (Biolegend, 69-0452-82, 0.2 μg/ml), APC-anti-CD138 (Biolegend, 142505, 0.2 μg/ml), PerCP/Cyanine5.5-anti-CD4 (Biolegend, 100434, 0.2 μg/ml), FITC-anti-CXCR5 (Biolegend, 1145519, 0.2 μg/ml), APC/Cyanine7-anti-PD-1 (Biolegend, 135223, 0.2 μg/ml), PE/Cyanine7-anti-Fas (Biolegend, 152617, 0.2 μg/ml), APC-anti-GL7 (Biolegend, 144617, 0.2 μg/ml), PE/Cyanine7-anti-CD25 (Biolegend, 102016, 0.2 μg/ml); For human samples, the cells were stained with Brilliant Violet 785-anti-CD3 (Biolegend, 300471, 0.2 μg/ml), PE/Cyanine7-anti-CD8a (301012, 0.2 μg/ml), Brilliant Violet 605-anti-CD19 (Biolegend, 302244, 0.2 μg/ml), Brilliant Violet 785-anti-CD38 (Biolegend, 303529, 0.2 μg/ml), APC-anti-CD20 (Biolegend, 302309, 0.2 μg/ml), APC-anti-CD4 (Biolegend, 344613, 0.2 μg/ml). And, the cells were analyzed using the BD LSRFortessa flow cytometer (BD Biosciences, San Jose, CA, USA).

For intracellular staining, the cells of mice were stimulated with 5 ng/mL phorbol myristate acetate (Sigma-Aldrich, St. Louis, MO, USA), 1 μg/mL brefeldin A (Sigma-Aldrich), and 500 ng/mL ionomycin (Sigma-Aldrich) at 37 °C for 4 h. After stimulation, the harvested cells of mice were fixed, permeabilized, stained with Brilliant Violet 650-anti-IL-17A (Biolegend, 506930, 0.3 μg/ml), PE-anti-IFN-γ (Biolegend, 505808, 0.3 μg/ml), FITC-anti-Foxp3 (Invitrogen, 11-5773-82, 0.3 μg/ml), APC-anti-phospho-AKT1 (Invitrogen, 17-9715-42, 0.5 µg/ml), anti-phospho-FOXO1 (Invitrogen, PA5-104977, 1:100), anti-PIM1 (NOVUS, NBP2-67528, 1:100), and anti-rabbit IgG (H + L), F(ab’)2 Fragment (Alexa Fluor 488 Conjugate) secondary antibody (Cell Signaling Technology, Danvers, MA, USA, 4412, 1:1,000). As for intracellular staining with human sample, the harvested cells were fixed, permeabilized, stained with anti-PIM1 (NOVUS, NBP2-67528, 1:100) and anti-rabbit IgG (H + L), F(ab’)2 Fragment (Alexa Fluor 488 Conjugate) secondary antibody (Cell Signaling Technology, 4412, 1:1,000). The cells were analyzed using flow cytometer and the results were analyzed using FlowJo software version 10.0.7 (BD Biosciences).

### Hematoxylin and eosin staining

After fixation and dehydration, mouse eyes were embedded in paraffin and sliced into 4 μm-thick sections. The sections were stained with hematoxylin and eosin, photographed, and pathological scores were assigned^64^. Histological evaluation was performed in a blinded manner. See Supplementary Table 1 for detailed scoring criteria.

### IRBP antibody assay

Serum levels of mouse antibodies against IRBP1-20 (IgG, IgG1, IgG2a, IgG2b, and IgG3) were quantified using ELISA. Briefly, after coating wells with 1 µg/mL IRBP1-20 overnight at 4 °C, non-specific binding was blocked with 3% bovine serum albumin (BSA) in PBS. Serially diluted serum samples were added to the wells for 1.5 h. Subsequently, after removing the serum and washing, bound antibodies against IRBP1-20 were detected with specific goat anti-mouse IgG (Southern Biotechnology, Birmingham, AL, USA). Absorbance was measured at 450 nm and the mean optical density (OD ± SEM) was calculated after adding stop solution.

### Immunofluorescence

After fixation and dehydration with graded sucrose solutions, CDLNs were embedded in Tissue-Tek O.C.T Compound (Sakura Finetek USA, Inc., Torrance, CA, USA). Sagittal sections of CDLNs (4 µm) were blocked with 3% BSA, stained with anti-PIM1 antibodies (NOVUS, NBP2-67528, 1:100) and anti-rabbit IgG (H + L), F(ab’)2 Fragment (Alexa Fluor 488 Conjugate) secondary antibody (Cell Signaling Technology, 4412, 1:1,000). Following staining with DAPI (Abcam), the sections were viewed under a fluorescence confocal microscope (ZEISS LSM 880, Germany) and photographed. The number of PIM1^+^ cells per unit area (mm^2^) (20×) was analyzed via ImageJ software v1.46r (National Institutes of Health, Bethesda, MD, USA) in a blinded manner.

### Functional assays

For PC differentiation, B cells of CDLNs were isolated using the mouse CD19 Positive Selection Kit II (STEMCELL, Vancouver, BC, Canada, 18954). Purified B cells were cultured with 10 ng/mL IL-4 (PeproTech, Rocky Hill, NJ, USA), 5 ng/mL IL-5 (PeproTech), 10 μg/mL F(ab′)2 goat anti-mouse IgM (Jackson ImmunoResearch, West Grove, PA, USA) and 80 ng/mL CD40L (PeproTech) in RPMI-1640 medium (Thermo Fisher Scientific, Waltham, MA, USA) supplemented with 10% fetal bovine serum (FBS), glutamine, 50 μM β-mercaptoethanol, and penicillin/streptomycin with SMI-4a or DMSO vehicle for 4 days at 37 °C.

### CFSE proliferation assessment

CD4^+^ T cells of PBMC were isolated using the Human CD4 Positive Selection Kit II (STEMCELL, 17852). Purified CD4^+^ T cells were conjugated with 2.5 mM CFSE (BD Biosciences) for 10 min at 37 °C while mixing continuously. Chilled complete growth media was added to quench the labeling reaction. Subsequently, the cells labeled with CFSE were stimulated with CD3/28 Dynabeads (GIBCO, Grand Island, NY, USA) or CD3/28 Dynabeads plus SMI-4a (20 μM). After incubation for 72 h, the proliferation rate of CD4^+^ T cells was measured using flow cytometry. Similarly, B cells sorted from PBMCs were conjugated with 2.5 mM CFSE. The cells labeled with CFSE were stimulated with CD40L and IL-4, or CD40L and IL-4 plus SMI-4a (20 μM). After incubation for 4 days, the proliferation rate of B cells was assessed using flow cytometry.

### Cell viability assay

Cell viability was determined using a CCK-8 assay kit (Dojindo Laboratories, Kumamoto, Japan) according to the manufacturer’s instructions. Briefly, 2 × 10^5^ cells treated with or without SMI-4a for 72 h were added to the wells of a 96-well plate. Samples were incubated with CCK-8 solution (10 μL) for 4 h at 37 °C. The absorbance of each well was measured at 450 nm using an automated enzyme-linked immunosorbent assay reader.

### Isolation of CDLNs for scRNA-seq

For pipeline analysis, different animals were used at different time points. CDLNs were harvested from mice in the day 0 group (*n* = 6), three control groups (days 7, 14, and 21; *n* = 3 at each time point), or three EAU groups (days 7, 14, and 21; *n* = 6 at each time point). CDLN cells harvested from three mice per group were pooled into one sample for single-cell library preparation. After grinding, pooled CDLN cells were mixed with RPMI-1640 medium containing 2% FBS (Thermo Fisher Scientific), penicillin/streptomycin (Thermo Fisher Scientific), 3 mg/mL collagenase IV (Sigma-Aldrich), and 40 mg/mL DNase I (Sigma-Aldrich), followed by incubation at 37 °C for 15 min. The digested cells were collected and filtered through a 70 mm cell strainer. The single-cell suspension contained 1 × 10^7^ cells/mL (viability ≥ 85%), as determined using the Countess II Automated Cell Counter (Thermo Fisher Scientific).

### Isolation of PBMCs for scRNA-seq

For pipeline assessment, we obtained venous blood samples from HCs and patients with VKH. Then the blood samples PBMCs were isolated using Ficoll-Hypaque solution (GE Healthcare, Chicago, IL, USA), followed by standard density gradient centrifugation. The cell viability was more than 85% in all the samples.

### scRNA-seq data processing

scRNA-seq libraries were generated using the Chromium Single Cell 5′ Library and Gel Bead Kit (10x Genomics, 120237) according to the manufacturer’s instructions, with some modifications. In detail, after washing with 0.04% BSA buffer (0.02 g BSA dissolved in 50 ml of deionized PBS), cells were captured in droplets. Then, reverse transcription, emulsion breaking, barcoded-cDNA purification with Dynabeads, and PCR amplification were conducted step by step. The amplified cDNA was then used to construct the 5′ gene expression library. Specifically, fragmenting and end-repair, double-size selection with SPRIselect beads, and sequencing were conducted on 50 ng of amplified cDNA using the NovaSeq platform (Illumina NovaSeq6000) to yield 150 bp paired-end reads. The number of reads and total genes detected in scRNAseq can be seen in Supplementary Table 2. Initial processing of the sequenced data was performed using CellRanger software v3.0.2 (10x Genomics). Data were integrated and clustered using the Seurat package (v3.1) in R (v4.0.0).

For quality control, the filter criteria was 300–4000 genes with < 15% mitochondrial genes. For human samples, we filtered out cells that highly expressed HBB, HBA1, and several light and heavy chain transcripts, which were considered to be populations contaminated with red blood cells. For mouse samples, we filtered out cells that highly expressed Hbb-a1 and Hbb-bs, which were recognized as red blood cells. The batch effect across different samples was removed using the Harmony package (v1.0) in R^68^.

### Dimensionality reduction and clustering analysis

For scRNA-seq data analysis, the data were normalized using the “NormalizeData” function in the Seurat package in R. Subsequently, the “FindClusters” function was used to cluster cells and the “RunTSNE” function was used to visualize the data with a 2-dimensional TSNE algorithm. Additionally, the “FindAllMarkers” function was used to generate marker genes of different clusters. DEGs of distinct cell types were generated using the “FindMarkers” function with Wilcoxon Rank Sum test and adjusted by Bonferroni correction. (|Log2 (fold change) | > 0.25 and adjusted *P* values < 0.05). The DEGs used in volcano plot can be seen in Supplementary Data 1.

### GO analysis

Gene ontology (GO) analysis was performed using the Metascape webtool (www.metascape.org)^69^. The p values of GO terms are calculated based on the accumulative hypergeometric distribution by Metascape webtool. Among the top 50 enriched GO terms across different cell types, 5–10 GO terms or pathways associated with diseases were shown with the pheatmap (v1.0.12) and ggplot2 package (v3.2.1) in R.

### BCR sequencing

To obtain isotype information of immunoglobin, BCR sequencing was performed. PCR amplification was done to enrich the full-length BCR V(D)J segments for the amplified cDNA from 5′ libraries with a Chromium Single-Cell V(D)J Enrichment kit (10 Genomics, 1000072). In detail, after washing with 0.04% BSA buffer, cells were captured in droplets. Then, reverse transcription, emulsion breaking, barcoded-cDNA purification with Dynabeads, and PCR amplification were conducted step by step. Amplified cDNA was then used for BCR enrichment. Specifically, V(D)J region-enriched libraries were size selected with SPRI beads and sequenced on an Illumina NovaSeq 6000 instrument using 150 paired-end reads. The BCR sequences of each B cell were clustered using the CellRanger vdj pipeline (v3.0.2). Briefly, barcode information containing the clonotype frequency and BCR diversity metric was obtained. The scRepertoire (v0.99.16) and Seurat packages in R were used to combine the BCR clonotypes with scRNA-seq data from the 10× Genomics CellRanger pipeline.

### Statistics

All experiments were repeated at least three times. GraphPad Prism Software v 8.0.2 (GraphPad, Inc., La Jolla, CA, USA) was employed for data analysis and presentation. The results are presented as the mean ± SEM. Statistical analysis was performed using an unpaired two-tailed Student’s *t*-test, one-way ANOVA, or two-way ANOVA. *P* < 0.05 were considered to be statistically significant. ns, non-significant; *P < 0.05; **P < 0.01; ***P < 0.001; and ****P < 0.0001.

### Reporting summary

Further information on research design is available in the Nature Research Reporting Summary linked to this article.

## Supplementary information

Supplementary Information

Description of Additional Supplementary Files

Supplementary Data 1

Reporting Summary

## Data Availability

The single-cell sequencing data and BCR sequencing data generated in this study was deposited in the Genome Sequence Archive (GSA) under project number (PRJCA004238) and GSA accession number (mouse data: CRA003777; human data: HRA002504). Source data are provided with this paper.

## Source data

Source Data

## Code availability

The data analysis pipeline in our study was described on the 10X Genomics (https://www.10xgenomics.com/) and Seurat official websites (https://github.com/satijalab/seurat).
