# Supplementary material for: Multicellular immune dynamics implicate PIM1 as a potential therapeutic target for uveitis
Source: Nat Commun. 2022 Oct 4;13:5866. doi: 10.1038/s41467-022-33502-7 (PMC9532430; doi:10.1038/s41467-022-33502-7)
Supplement: Supplementary file 1 — Supplementary Information [file 41467_2022_33502_MOESM1_ESM.pdf]

Supplementary Information

Multicellular immune dynamics implicate PIM1 as a potential therapeutic target for uveitis

He Li[1, #], Lihui Xie[1, #], Lei Zhu[1, #], Zhaohuai Li[1, #], Rong Wang[1, #], Xiuxing Liu[1], Zhaohao Huang[1], Binyao Chen[1], Yuehan Gao[1], Lai Wei[1], Chang He[1], Rong Ju[1], Yizhi Liu[1, 2], Xialin Liu[1, *], Yingfeng Zheng[1, 2, *], Wenru Su[1, *]

[1]State Key Laboratory of Ophthalmology, Zhongshan Ophthalmic Center, Sun Yat-sen University, Guangdong Provincial Key Laboratory of Ophthalmology and Visual Science, Guangzhou 510060，China.

[2]Research Unit of Ocular Development and Regeneration, Chinese Academy of Medical Sciences, Beijing 100085, China

[#]These authors contributed equally

*Correspondence: Xialin Liu, liuxl28@mail.sysu.edu.cn; Yingfeng Zheng, zhyfeng@mail.sysu.edu.cn; Wenru Su, suwr3@mail.sysu.edu.cn

Supplementary Figures

Supplementary Figure 1

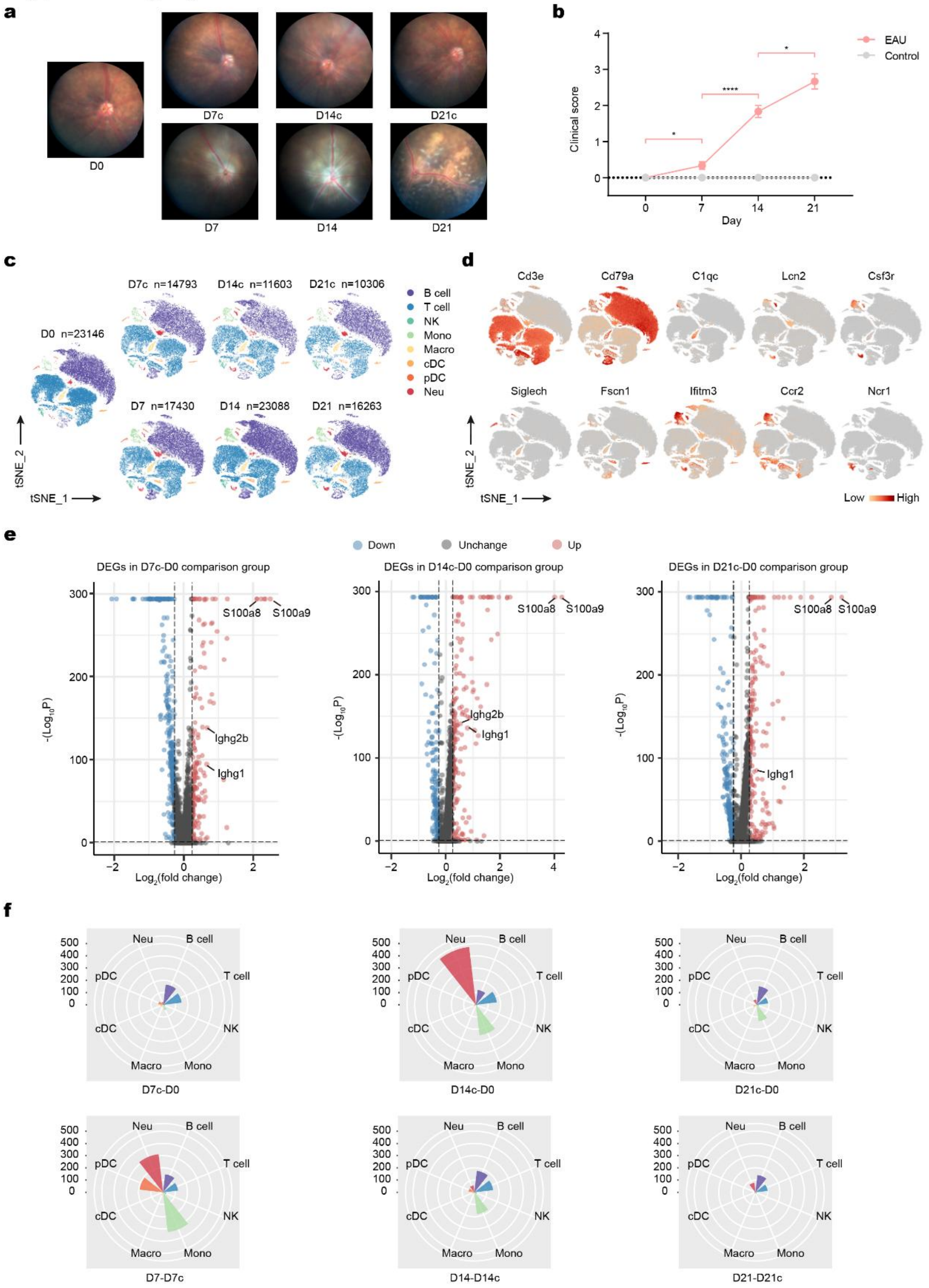

Supplementary Figure 1. Clinical scores, Clusters of major immune cell populations and analysis of gene expression alterations in EAU

(**a**) Representative fundus images of eyes from the day0 (D0), control (D7c, D14c, D21c) and EAU (D7, D14, D21) groups.
(**b**) Clinical scores of eyes from the control group and EAU group after immunization at day0, day7, day14 and day21. Each group contains six mice. *P*(D0-D7) = 0.0101, *P*(D7-D14) = 1.8E-05, *P*(D14-D21) = 0.0112. Data represented as mean ± SEM. Significance was determined using one-way ANOVA. *$P < 0.05$, ****$P < 0.0001$.
(**c**) t-SNE plots segregated by different time point and conditions (Day 0 group (D0), EAU groups (D7, D14, D21) and control group (D7c, D14c, D21c)).
(**d**) t-SNE plots of canonical markers for major immune cell types from all mice groups.
(**e**) Volcano plots showing upregulated and downregulated DEGs of total immune cells in the control groups at different time points compared to day 0 group. Red and blue dots indicate upregulated and downregulated DEGs in control groups compared to day 0 group, respectively. Significance was determined using "FindMarkers" functions of Seurat package with Wilcoxon Rank Sum test and adjusted by Bonferroni correction.
(**f**) Rose plots showing the number of DEGs of each immune cell type in the control/day 0 comparison groups and EAU/control comparison groups at different time points.

Supplementary Figure 2

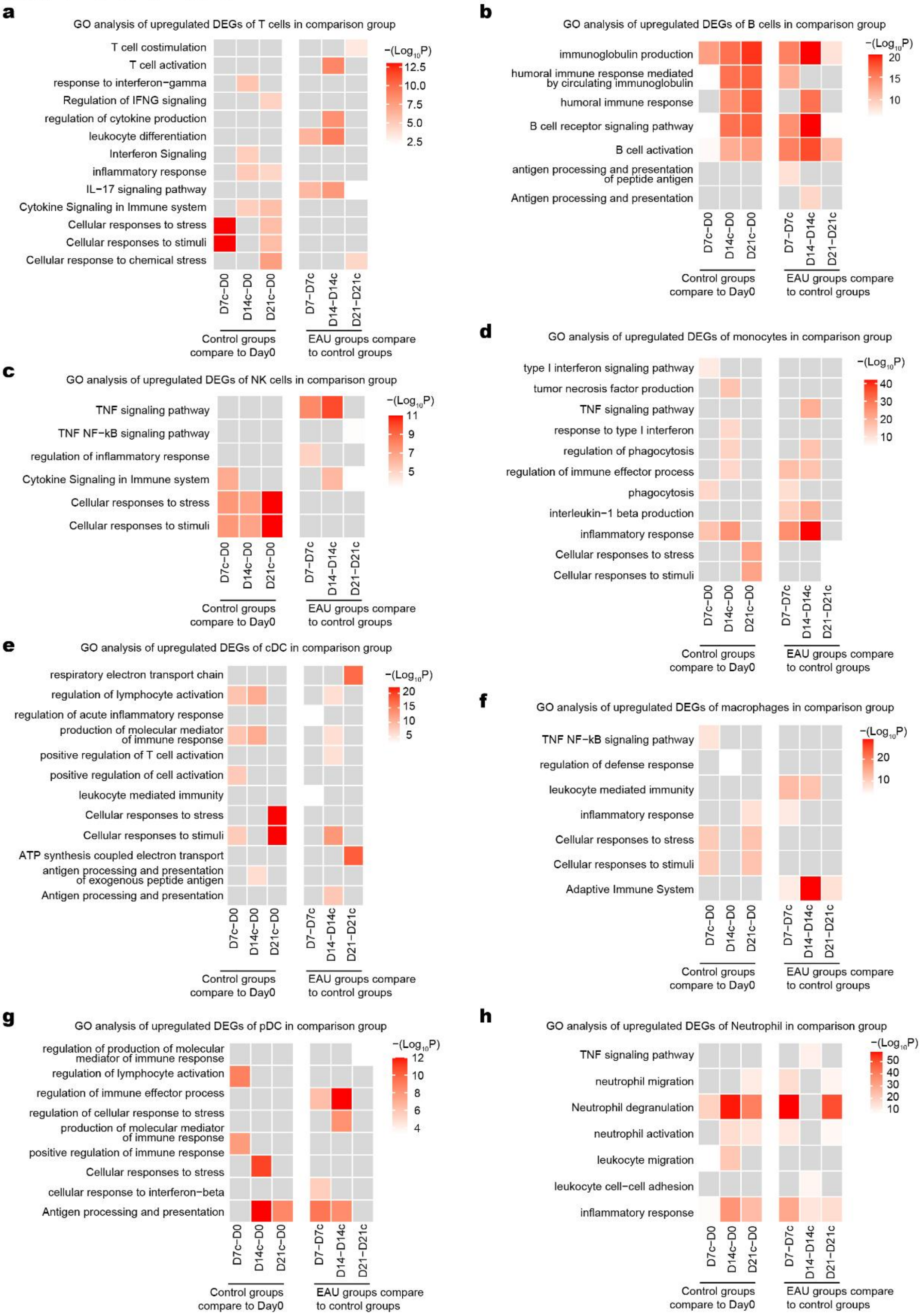

Supplementary Figure 2. GO analysis of the major immune cell types in EAU at different time points

(**a-h**) Heatmap showing representative GO terms and KEGG pathways enriched in upregulated DEGs of each major cell types in the control/day 0 comparison groups and EAU

/control comparison groups at different time points. Significance was calculated based on the accumulative hypergeometric distribution by Metascape webtool.

Supplementary Figure 3

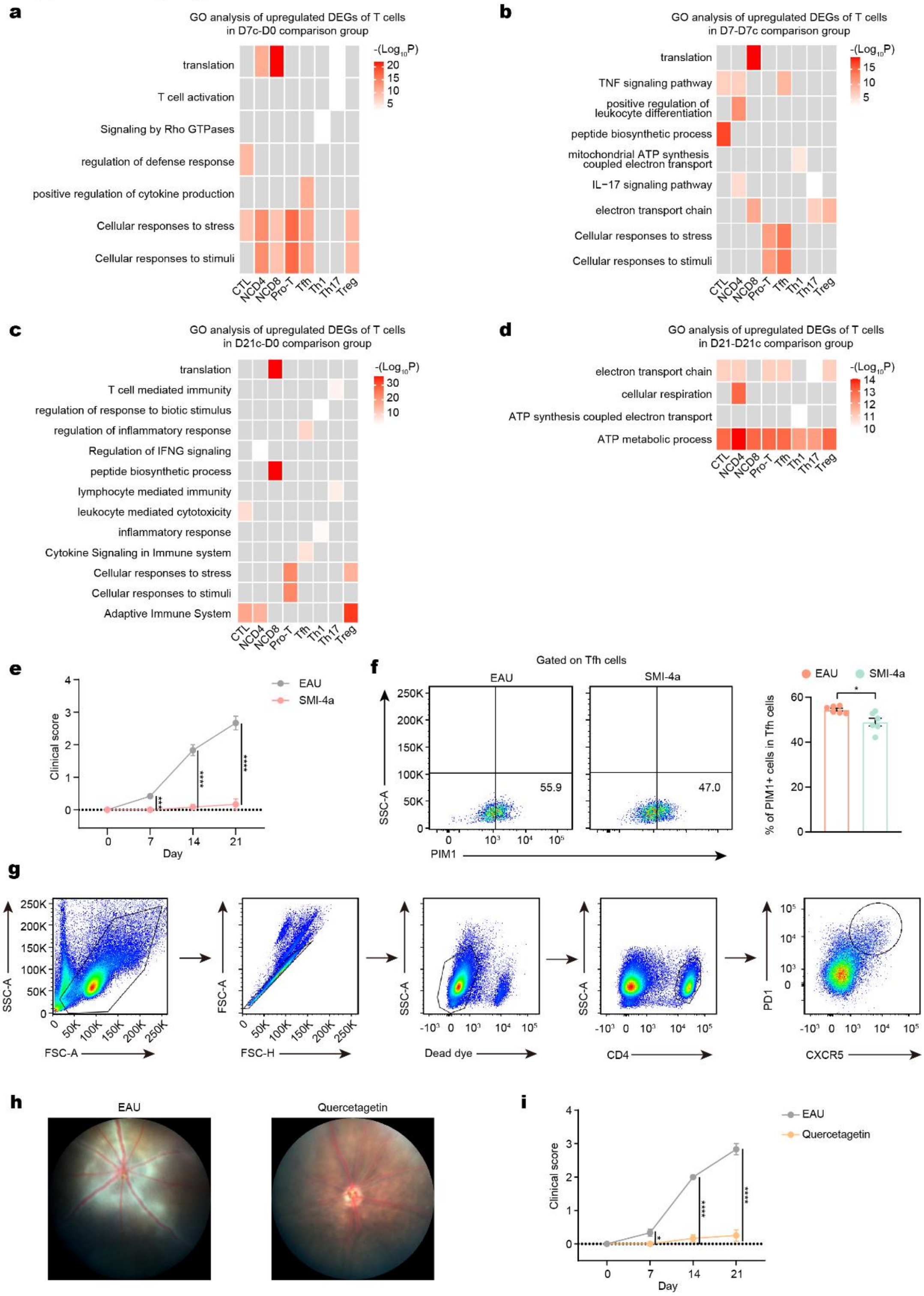

Supplementary Figure 3. GO analysis of the T cell subtypes in EAU at different time points

(**a-d**) Heatmap showing representative GO terms and KEGG pathways enriched in upregulated DEGs of the T cell subtypes in the control/day 0 comparison groups and

EAU/control comparison groups at different time points. Significance was calculated based on the accumulative hypergeometric distribution by Metascape webtool.

(**e**) Clinical scores of eyes from the vehicle group and SMI-4a group after immunization. Each group contains six mice. *P*(D7) = 0.0005, *P*(D14) = 2.8E-06, *P*(D21) = 3.1E-06. Data represented as mean ± SEM. Significance was determined using one-way ANOVA. ****P* < 0.001, *****P* < 0.0001.

(**f**) Proportions of $PIM1^+$ cells in Tfh cells were measured by flow cytometry after immunization at day 14. Each group contains six mice. *P*(EAU-SMI-4a) = 0.0147. Data expressed as mean ± SEM. Significance was determined using unpaired two-tailed student's t test. *****P* < 0.0001.

(**g**) Gating strategy of Tfh cells for supplementary figure 3f.

(**h**) Representative fundus images of eyes from the vehicle group and quercetagetin group after immunization at day 14.

(**i**) Clinical scores of eyes from the vehicle group and quercetagetin group after immunization. Each group contains six mice. *P*(D7) = 0.0101, *P*(D14) = 8.4E-09, *P*(D21) = 7.7E-07. Data represented as mean ± SEM. Significance was determined using one-way ANOVA. ns, no significant differences, **P* < 0.05, *****P* < 0.0001.

Supplementary Figure 4

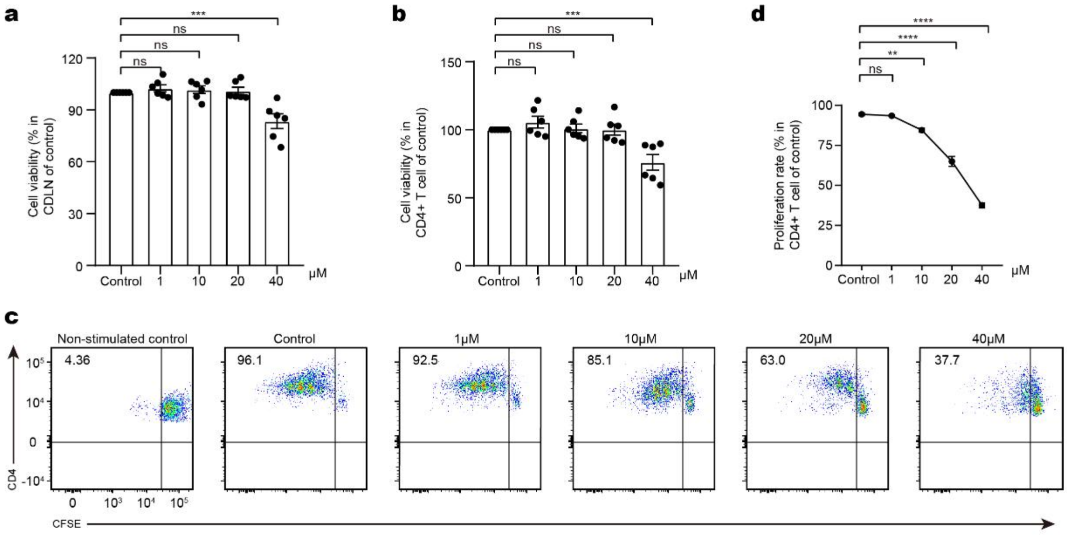

Supplementary Figure 4. Cytotoxicity of SMI-4a in mice

(**a**) CCK8 assay of CDLN cells treated by escalating doses of SMI-4a (0-40 μM) for 72 h. Data was shown as mean ± SEM from six independent experiments. *P*(Control-1μM) = 0.8912, *P*(Control-10μM) = 0.9646, *P*(Control-20μM) = 0.9950, *P*(Control-40μM) = 0.0004. Significance was determined using one-way ANOVA. ns, no significant differences, ****P* < 0.001.

(**b**) CCK8 assay of $CD4^+$ T cells isolated from CDLN cells treated by escalating doses of SMI-4a (0-40 μM) for 72 h. Data was shown as mean ± SEM from six independent experiments. *P*(Control-1μM) = 0.7083, *P*(Control-10μM) = 0.9991, *P*(Control-20μM) = 1.0000, *P*(Control-40μM) = 0.0009. Significance was determined using one-way ANOVA. ns, no significant differences, ****P* < 0.001.

(**c-d**) Proliferation rate of $CD4^+$ T cells treated by escalating doses of SMI-4a (0-40 μM) for 72 h. Proliferation rate was measured by flow cytometry. Data was shown as mean ± SEM from six independent experiments. *P*(Control-1μM) = 0.9867, *P*(Control-10μM) = 0.0010, *P*(Control-20μM) = 1.0E-15, *P*(Control-40μM) = 1.0E-15. Significance was determined using one-way ANOVA. ns, no significant differences, ***P* < 0.01, *****P* < 0.0001.

Supplementary Figure 5

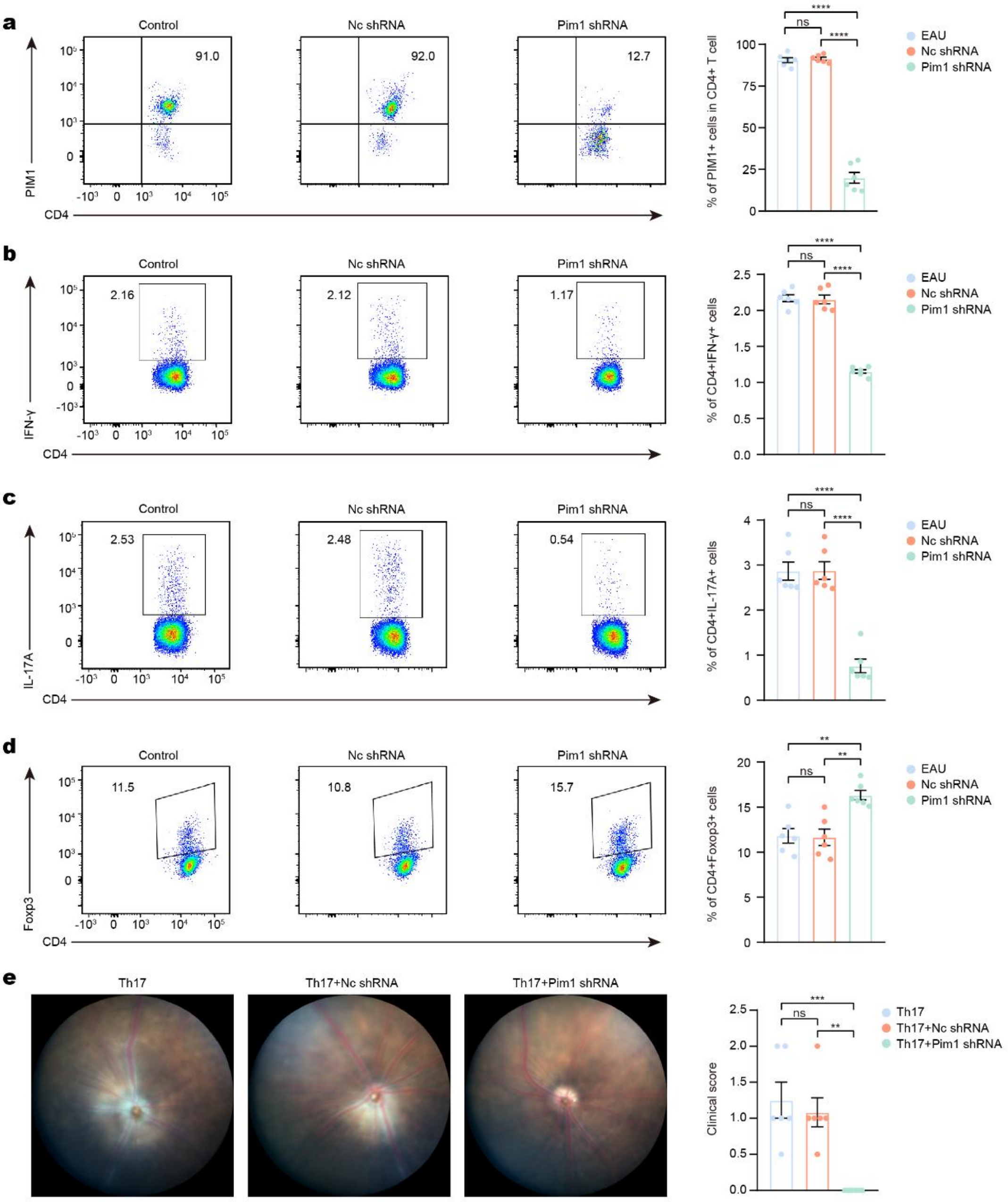

Supplementary Figure 5. Pim1 shRNA regulate Th17/Treg balance

(**a**) After treated with Pim1 shRNA or negative control (nc) shRNA, flow cytometry was performed to show the proportion of PIM1^{+} cells in total CD4^{+} cells. Each group contains six mice. Data expressed as mean ± SEM. *P*(Control-Nc shRNA) = 0.9581, *P*(Control-pim1

shRNA) = 6.9E-13, *P*(Nc shRNA- pim1 shRNA) = 4.8E-13. Significance was determined using one-way ANOVA. ns, no significant differences, ****$P < 0.0001$.

(**b-d**) After treated with Pim1 shRNA or negative control (nc) shRNA, CDLN cells from EAU mice were cultured with IRBP1-20. Flow cytometry was performed to show the proportion of Th1 cells (**b**), Th17 cells (**c**) and Treg cells (**d**). Each group contains six mice. *P*(Th1 cells, Control-Nc shRNA) = 0.9589, *P*(Th1 cells, Control-pim1 shRNA) = 4.0E-10, *P*(Th1 cells, Nc shRNA- pim1 shRNA) = 5.2E-10, *P*(Th17 cells, Control-Nc shRNA) = 0.9985, *P*(Th17 cells, Control-pim1 shRNA) = 2.1E-06, *P*(Th17 cells, Nc shRNA- pim1 shRNA) = 1.9E-06, *P*(Treg, Control-Nc shRNA) = 0.9870, *P*(Treg, Control-pim1 shRNA) = 0.0022, *P*(Treg, Nc shRNA-pim1 shRNA) = 0.0016. Data expressed as mean ± SEM. Significance was determined using one-way ANOVA. ns, no significant differences, **$P < 0.01$, ****$P < 0.0001$.

(**e**) Representative fundus images and clinical score after induction of Pim1 shRNA treated-Th17 cells ($CD4^+CCR6^+CXCR3^-$ cells) cultured with IRBP1-20 at day 14. Each group contains six mice. *P*(Th17) = 0.9552, *P*(Th17+Nc shRNA) = 0.0002, *P*(Th17+pim1 shRNA) = 0.0003. Data expressed as mean ± SEM. Significance was determined using one-way ANOVA. ns, no significant differences, **$P < 0.01$, ***$P < 0.001$.

Supplementary Figure 6

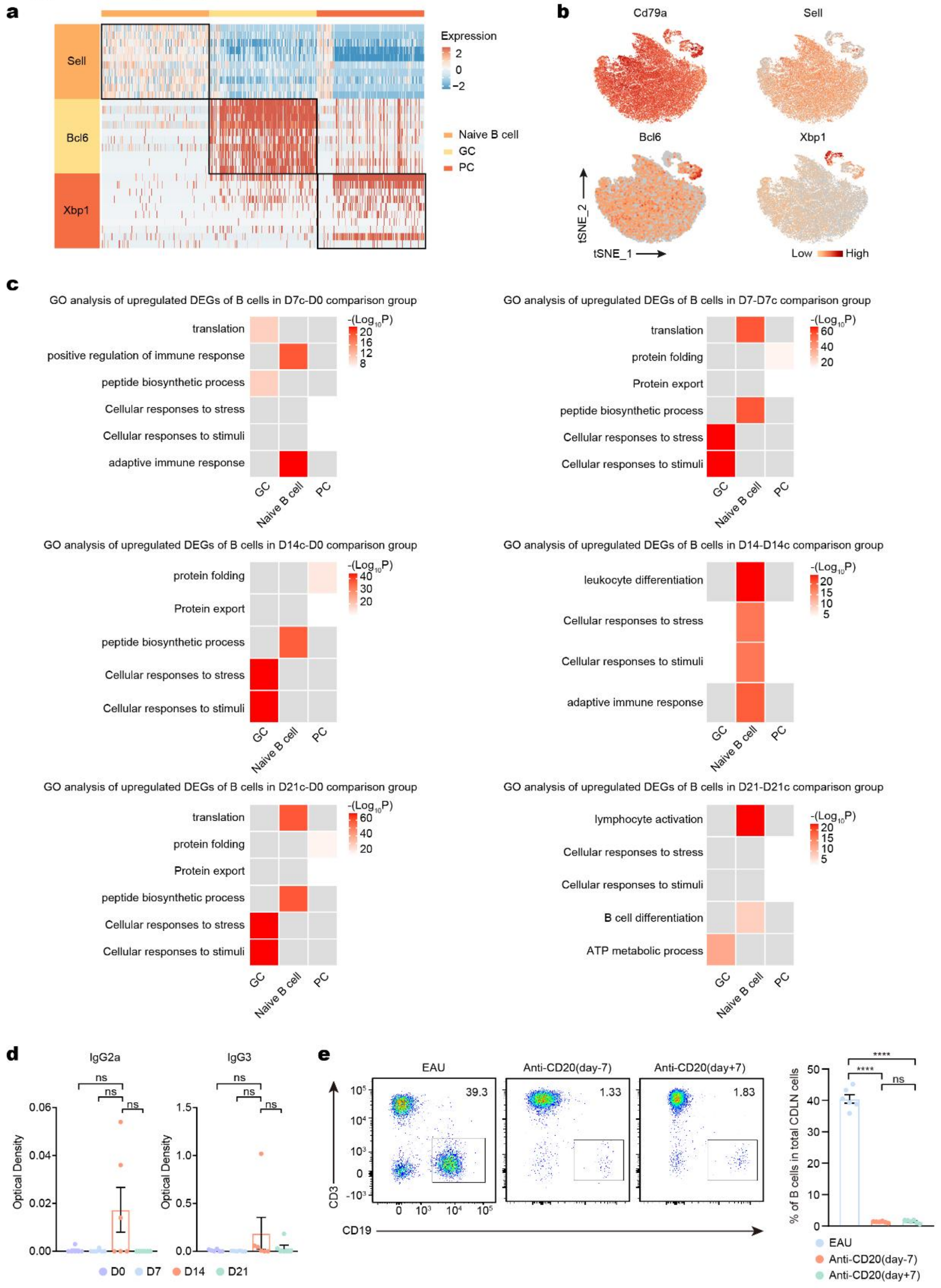

Supplementary Figure 6. Dynamic changes in B cell subsets during EAU

(**a**) Heatmap showing scaled expression of discriminative gene sets for B cell subsets from all mice groups.

(**b**) t-SNE plots of canonical markers for B cell subsets from all mice groups.

(**c**) Heatmap showing representative GO terms and KEGG pathways enriched in upregulated DEGs of the B cell subsets in the control/day 0 comparison groups and EAU/control comparison groups at different time points. Significance was calculated based on the accumulative hypergeometric distribution by Metascape webtool.
(**d**) Concentration of IRBP1-20-specific antibodies (IgG2a and IgG3) in the blood serum from day 0 group and EAU groups at different time point, detected by ELISA. Each group contains six mice. *P*(IgG2a, D0-D14) = 0.0822, *P*(IgG2a, D7-D14) = 0.0763, *P*(IgG2a,D14-D21) = 0.0708, *P*(IgG3, D0-D14) = 0.4619, *P*(IgG3, D7-D14) = 0.4284, *P*(IgG3,D14-D21) = 0.5870. Data represented as mean ± SEM. Significance was determined using one-way ANOVA. ns, no significant differences.
(**e**) Administration of anti-CD20 antibodies to deplete the B cells before (7 days before immunization, day −7) and after EAU development (7 days after immunization, day +7). Proportions of B cells in CDLN cells were measured by flow cytometry after immunization at day 14. Each group contains six mice. *P*(EAU- Anti-CD20 (day-7)) = 1.0E-15, *P*(EAU- Anti-CD20 (day+7)) = 1.0E-15, *P*(Anti-CD20 (day-7)-Anti-CD20 (day+7)) = 0.9929. Data expressed as mean ± SEM. Significance was determined using one-way ANOVA. ns, no significant differences, ****$P < 0.0001$.

Supplementary Figure 7

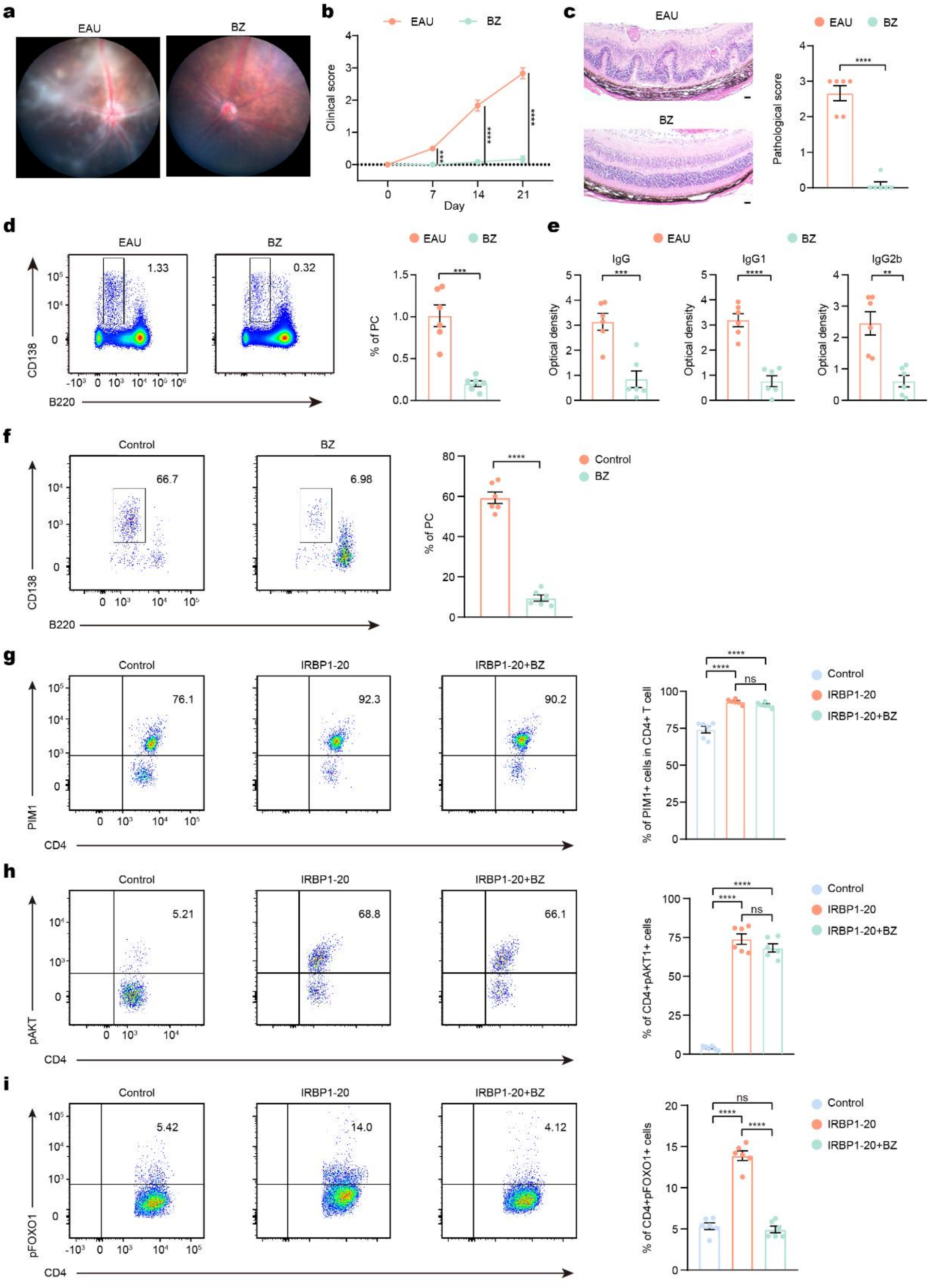

Supplementary Figure 7. Bortezomib (BZ) alleviated EAU

(**a**) Representative fundus images of eyes from EAU mice treated with BZ or vehicle obtained 14 days after immunization.

(**b**) Clinical scores of eyes from EAU mice treated with BZ or vehicle after immunization. Each group contains six mice. *P*(D7) = 0.0005, *P*(D14) = 6.6E-07, *P*(D21) = 9.4E-08. Data

expressed as mean ± SEM. Significance was determined using one-way ANOVA. ****P* < 0.001, *****P* < 0.0001.

(**c**) Representative histopathological images (hematoxylin and eosin staining) and pathogenic scores of eyes from EAU mice treated with BZ or vehicle obtained 14 days after immunization. Each group contains six mice. *P*(EAU-BZ) = 4.7E-07. Data expressed as mean ± SEM. Significance was determined using unpaired two-tailed student's t test. *****P* < 0.0001. Scale bars, 20 mm.

(**d**) Frequency of plasma cells from EAU mice treated with BZ or vehicle. Cells were gated on the $CD45^{+}CD90.2^{-}CD11c^{-}F480^{-}$ population. Each group contains six mice. *P*(EAU-BZ) = 0.0001. Data represented as mean ± SEM. Significance was determined using unpaired two-tailed student's t test. ****P* < 0.001.

(**e**) Concentration of IRBP1-20-specific antibodies in EAU mice treated with BZ or vehicle. Each group contains six mice. *P*(IgG) = 0.0007, *P*(IgG1) = 2.9E-05, *P*(IgG2b) = 0.0012. Data represented as mean ± SEM. Significance was determined using unpaired two-tailed student's t test. ***P* < 0.01, ****P* < 0.001, and *****P* < 0.0001.

(**f**) Sorted B cells were stimulated with anti-IgM F(ab)2, CD40L, IL-4, and IL-5 for 5 days with or without BZ (1 nM). The proportion of plasma cells was measured by flow cytometry. Data represented as mean ± SEM from six independent experiments. *P*(Control-BZ) = 2.8E-08. Significance was determined using unpaired two-tailed student's t test. *****P* < 0.0001.

(**g-i**) $CD4^{+}$ T cells from EAU group cultured with IRBP1-20 alone or with IRBP1-20 plus with BZ (1 nM) for 72h. Flow cytometry showed the proportion of $PIM1^{+}$ cells (**g**), $pAKT^{+}$ cells (**h**), and $pFOXO1^{+}$ cells (**i**) in $CD4^{+}$ T cells. Data represented as mean ± SEM from six independent experiments. *P*($PIM1^{+}$ cells,Control-IRBP1-20) = 4.2E-07, *P*($PIM1^{+}$ cells, Control-IRBP1-20+BZ) = 1.9E-06, *P*($PIM1^{+}$ cells, IRBP1-20-IRBP1-20+BZ) = 0.5621, *P*($pAKT^{+}$ cells,Control-IRBP1-20) = 8.5E-12, *P*($pAKT^{+}$ cells, Control-IRBP1-20+BZ) = 2.7E-11, *P*($pAKT^{+}$ cells, IRBP1-20-IRBP1-20+BZ) = 0.2546, *P*($pFOXO1^{+}$ cells,Control-IRBP1-20) = 4.7E-09, *P*($pFOXO1^{+}$ cells, Control-IRBP1-20+BZ) = 0.8135, *P*($pFOXO1^{+}$ cells, IRBP1-20-IRBP1-20+BZ) = 2.5E-09. Significance was determined using two-way ANOVA. ns, no significant differences, *****P* < 0.0001.

Supplementary Figure 8

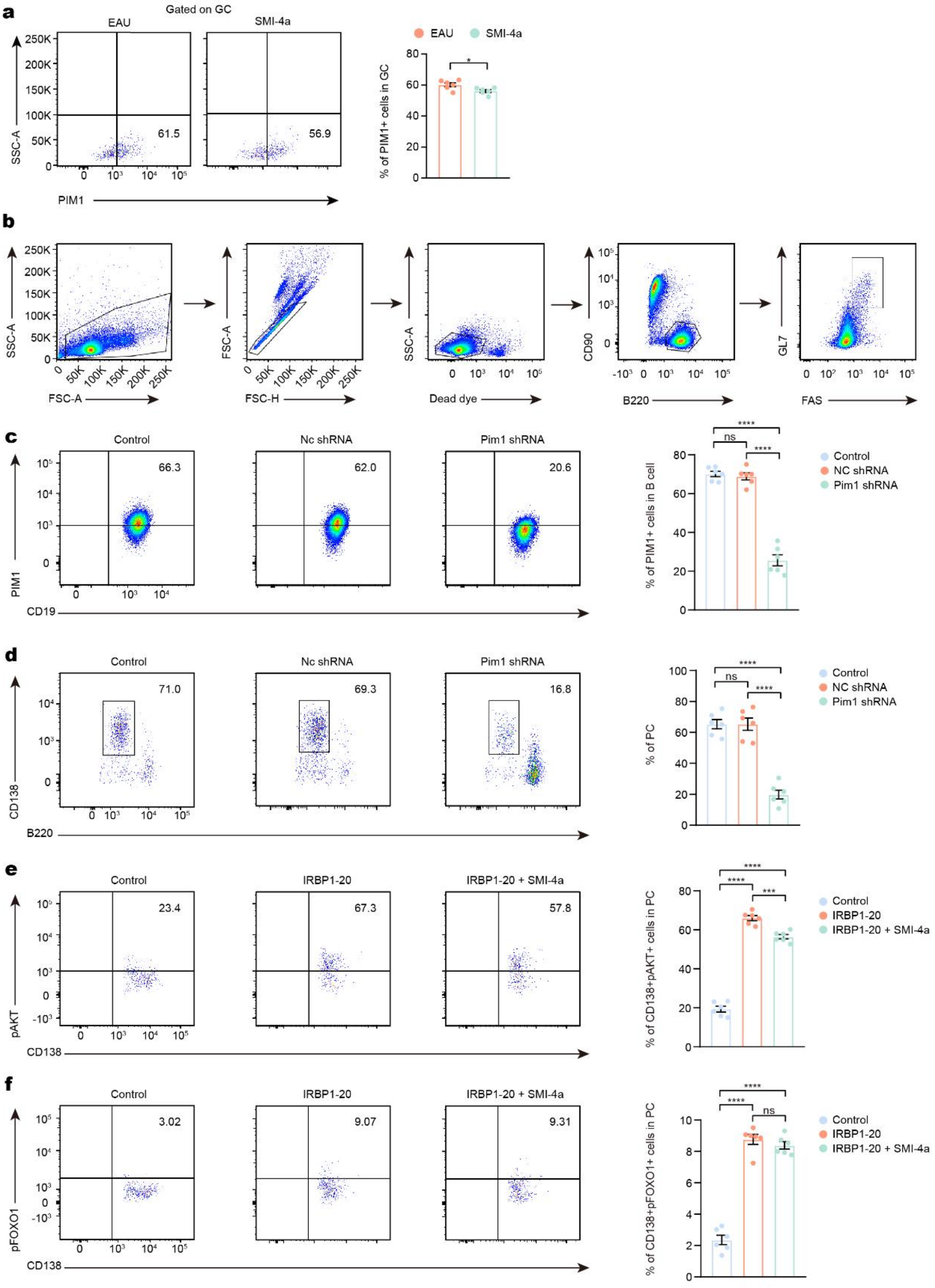

Supplementary Figure 8. Bortezomib (BZ) alleviated EAU

(**a**) Proportions of PIM1+ cells in GC cells were measured by flow cytometry after immunization at day 14. Each group contains six mice. *P*(EAU-SMI-4a) = 0.0175. Data expressed as mean ± SEM. Significance was determined using unpaired two-tailed student's t test. $^{*}P < 0.05$.

(**b**) Gating strategy of GC cells for supplementary figure 8a.
(**c**) After treated with Pim1 shRNA or negative control (nc) shRNA, the proportion of $PIM1^+$ cells in B cells was measured by flow cytometry. Each group contains six mice. *P*(Control-Nc shRNA) = 0.9134, *P*(Control-pim1 shRNA) = 6.2E-10, *P*(Nc shRNA-pim1 shRNA) = 9.3E-10. Data expressed as mean ± SEM. Significance was determined using one-way ANOVA. ns, no significant differences, ****$P < 0.0001$.
(**d**) Sorted B cells treated with or Pim1 shRNA or nc shRNA were stimulated with anti-IgM F(ab)2, CD40L, IL-4, and IL-5 for 5 days. The proportion of plasma cells was measured by flow cytometry. Data represented as mean ± SEM from six independent experiments. *P*(Control-Nc shRNA) = 1.0000, *P*(Control-pim1 shRNA) = 2.2E-07, *P*(Nc shRNA- pim1 shRNA) = 2.2E-07. Significance was determined using one-way ANOVA. ns, no significant differences, ****$P < 0.0001$.
(**e-f**) Plasma cells from EAU group cultured with IRBP1-20 alone or with IRBP1-20 plus with SMI-4a for 72h. Flow cytometry showed the proportion of $pAKT^+$ cells (**e**), and $pFOXO1^+$ cells (**f**) in PC. Data represented as mean ± SEM from six independent experiments. *P*($pAKT^+$ cells,Control-IRBP1-20) = 1.0E-15, *P*($pAKT^+$ cells, Control-IRBP1-20+SMI-4a) = 8.6E-12, *P*($pAKT^+$ cells, IRBP1-20-IRBP1-20+ SMI-4a) = 0.0003, *P*($pFOXO1^+$ cells,Control-IRBP1-20) = 3.0E-05, *P*($pFOXO1^+$ cells, Control-IRBP1-20+SMI-4a) = 4.7E-05, *P*($pFOXO1^+$ cells, IRBP1-20-IRBP1-20+SMI-4a) = 0.9591. Significance was determined using two-way ANOVA. ns, no significant differences, ****$P < 0.0001$.

Supplementary Figure 9

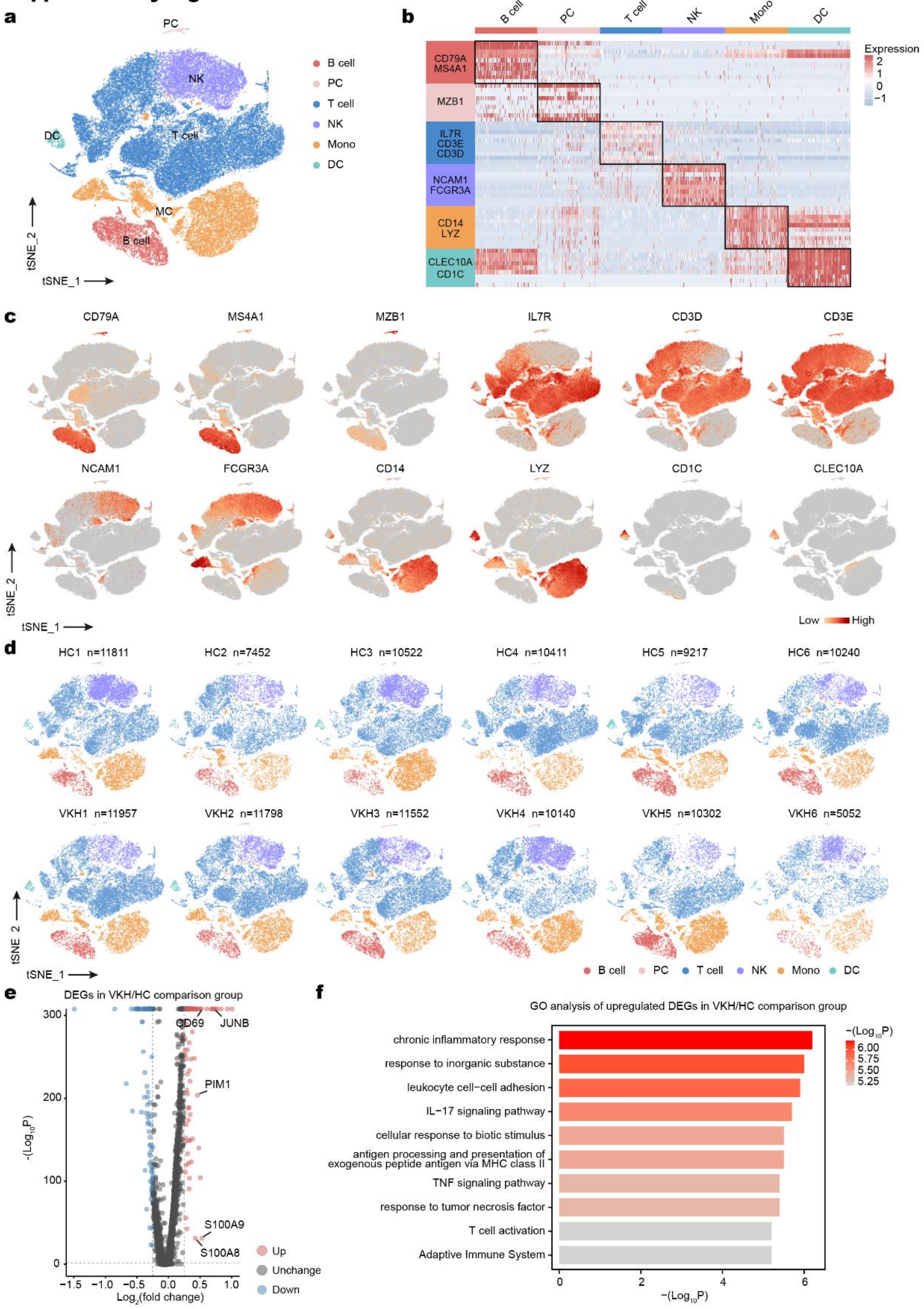

Supplementary Figure 9. Clusters of major immune cell populations and analysis of gene expression alterations in VKH

(**a**) t-SNE plots of major immune cell types from all HC and VKH samples.

(**b**) Heatmap showing scaled expression of discriminative gene sets for each immune cell type from all HC and VKH samples.

(**c**) t-SNE plots of canonical markers for the major immune cell types from all HC and VKH samples.
(**d**) t-SNE plots segregated by different individuals.
(**e**) Volcano plots showing upregulated and downregulated DEGs of total immune cells in the VKH/HC comparison groups. Red and blue dots indicate upregulated and downregulated DEGs in VKH groups compared to HC group, respectively. Significance was determined using "FindMarkers" functions of Seurat package with Wilcoxon Rank Sum test and adjusted by Bonferroni correction.
(**f**) Bar chart showing representative GO terms and KEGG pathways enriched in upregulated DEGs of total immune cells in the VKH/HC comparison groups. Significance was calculated based on the accumulative hypergeometric distribution by Metascape webtool.

Supplementary Figure 10

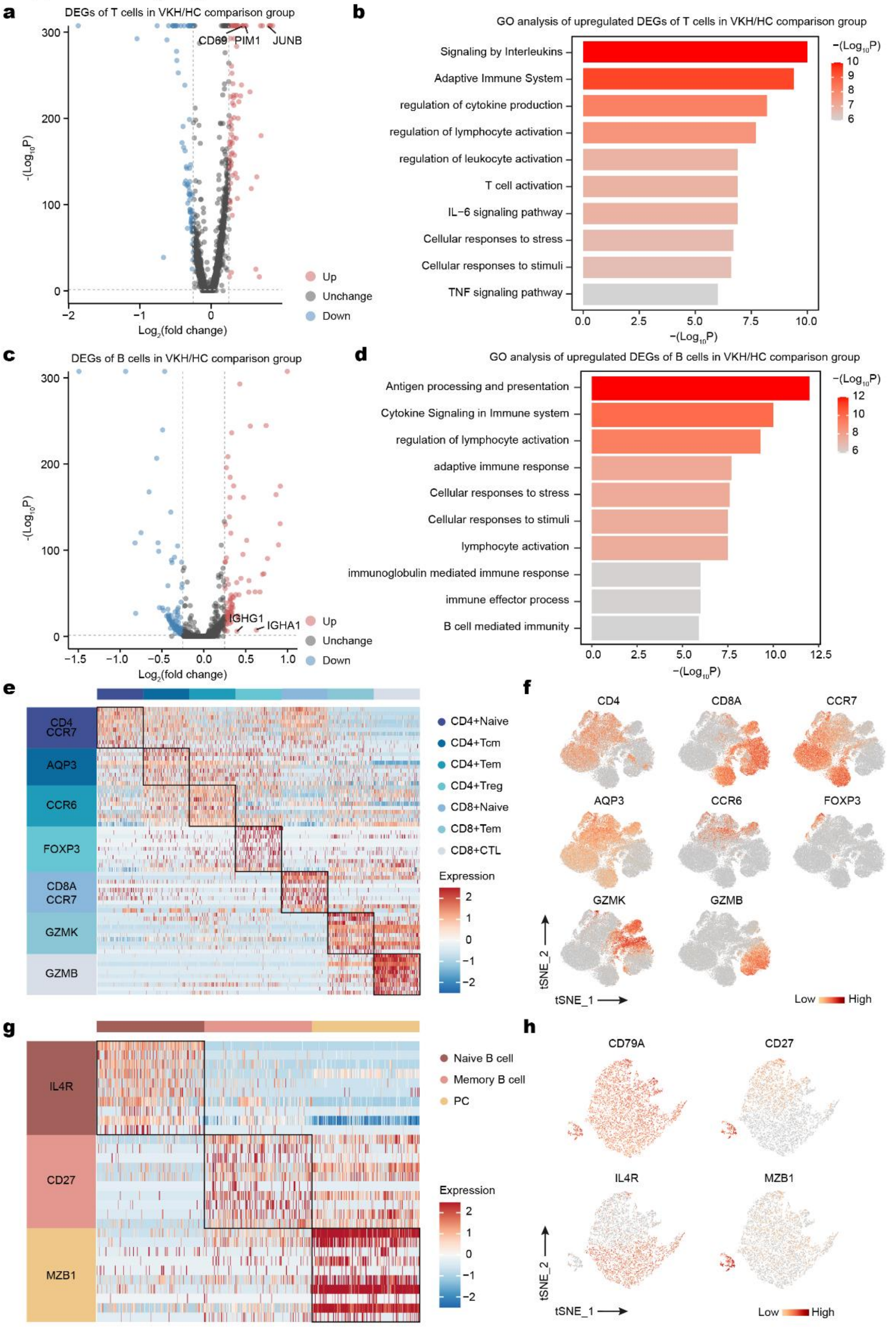

Supplementary Figure 10. Analysis of gene expression alterations of T and B cells and clustering their subsets in VKH

(**a**) Volcano plots showing upregulated and downregulated DEGs of T cells in the VKH/HC comparison groups. Red and blue dots indicate upregulated and downregulated DEGs in VKH groups compared to HC group, respectively. Significance was determined using

“FindMarkers” functions of Seurat package with Wilcoxon Rank Sum test and adjusted by Bonferroni correction.

(**b**) Bar chart showing representative GO terms and KEGG pathways enriched in upregulated DEGs of T cells in the VKH/HC comparison groups. Significance was calculated based on the accumulative hypergeometric distribution by Metascape webtool.

(**c**) Volcano plots showing upregulated and downregulated DEGs of B cells in the VKH/HC comparison groups. Red and blue dots indicate upregulated and downregulated DEGs in VKH groups compared to HC group, respectively. Significance was determined using “FindMarkers” functions of Seurat package with Wilcoxon Rank Sum test and adjusted by Bonferroni correction.

(**d**) Bar chart showing representative GO terms and KEGG pathways enriched in upregulated DEGs of B cells in the VKH/HC comparison groups. Significance was calculated based on the accumulative hypergeometric distribution by Metascape webtool.

(**e**) Heatmap showing scaled expression of discriminative gene sets for T cell subsets from all HC and VKH samples.

(**f**) t-SNE plots of canonical markers for T cell subsets from all HC and VKH samples.

(**g**) Heatmap showing scaled expression of discriminative gene sets for B cell subsets from all HC and VKH samples.

(**h**) t-SNE plots of canonical markers for B cell subsets from all HC and VKH samples.

Supplementary Figure 11

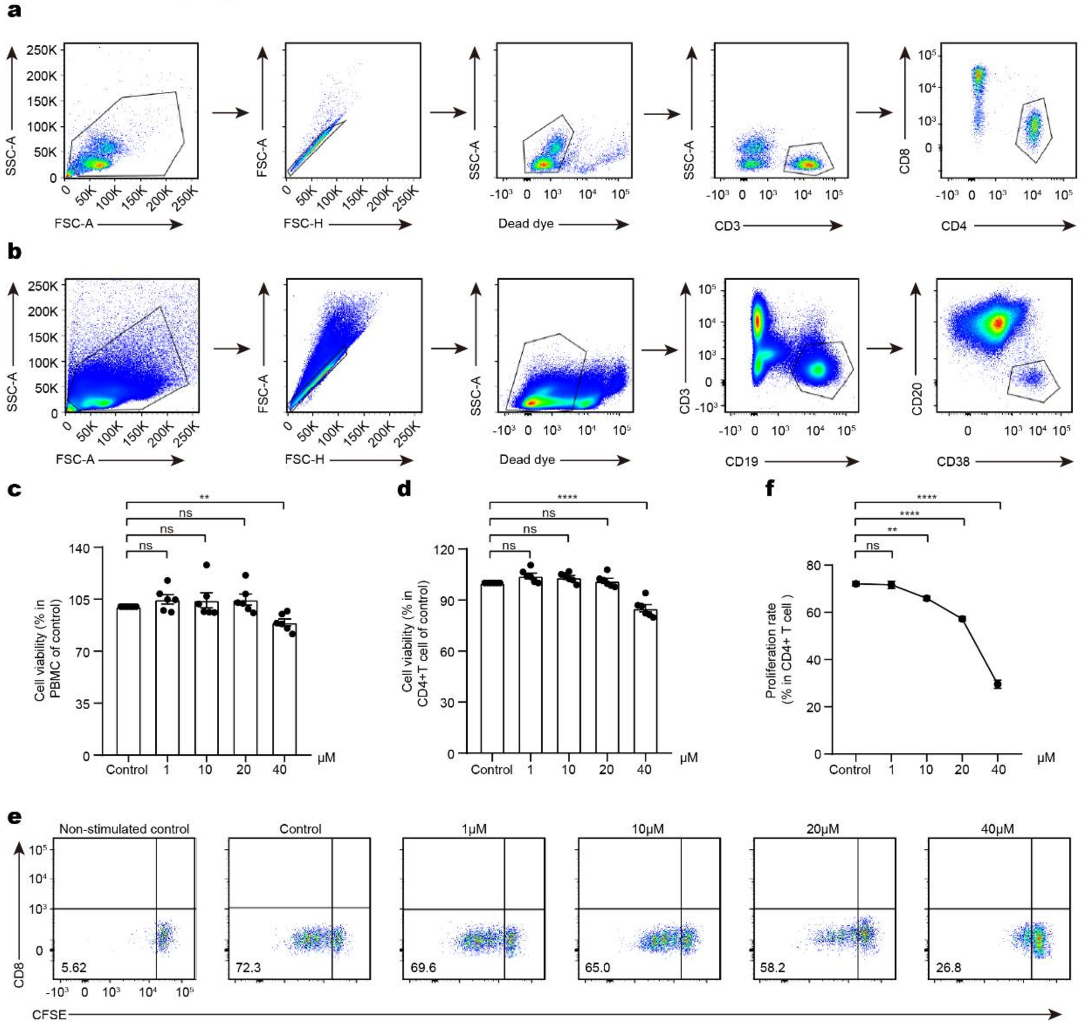

Supplementary Figure 11. Cytotoxicity of SMI-4a in human

(**a**) Gating strategy of CD4⁺ T cells for figure 6f.

(**b**) Gating strategy of plasma cells for figure 6g.

(**c**) CCK8 assay of PBMC cells treated by escalating doses of SMI-4a (0-40 μM) for 72 h. Data was shown as mean ± SEM from six independent experiments. *P*(Control-1μM) = 0.6532,

P(Control-10μM) = 0.7414, *P*(Control-20μM) = 0.6706, *P*(Control-40μM) = 0.0104. Significance was determined using one-way ANOVA. ns, no significant differences, $^{***}P < 0.001$.

(**d**) CCK8 assay of $CD4^+$ T cells isolated from PBMC cells treated by escalating doses of SMI-4a (0-40 μM) for 72 h. Data was shown as mean ± SEM from six independent experiments. *P*(Control-1μM) = 0.1462, *P*(Control-10μM) = 0.3257, *P*(Control-20μM) = 0.9162, *P*(Control-40μM) = 1.0E-15. Significance was determined using one-way ANOVA. ns, no significant differences, $^{****}P < 0.0001$.

(**e-f**) Proliferation rate of $CD4^+$ T cells (gated on $CD3^+CD8^-$ T cells) treated by escalating doses of SMI-4a (0-40 μM) for 72 h. Proliferation rate was measured by flow cytometry. Data was shown as mean ± SEM from six independent experiments. *P*(Control-1μM) = 0.9974, *P*(Control-10μM) = 0.0073, *P*(Control-20μM) = 1.0E-15, *P*(Control-40μM) = 1.0E-15. Significance was determined using one-way ANOVA. ns, no significant differences, $^{**}P < 0.01$, $^{****}P < 0.0001$.

Supplementary Figure 12

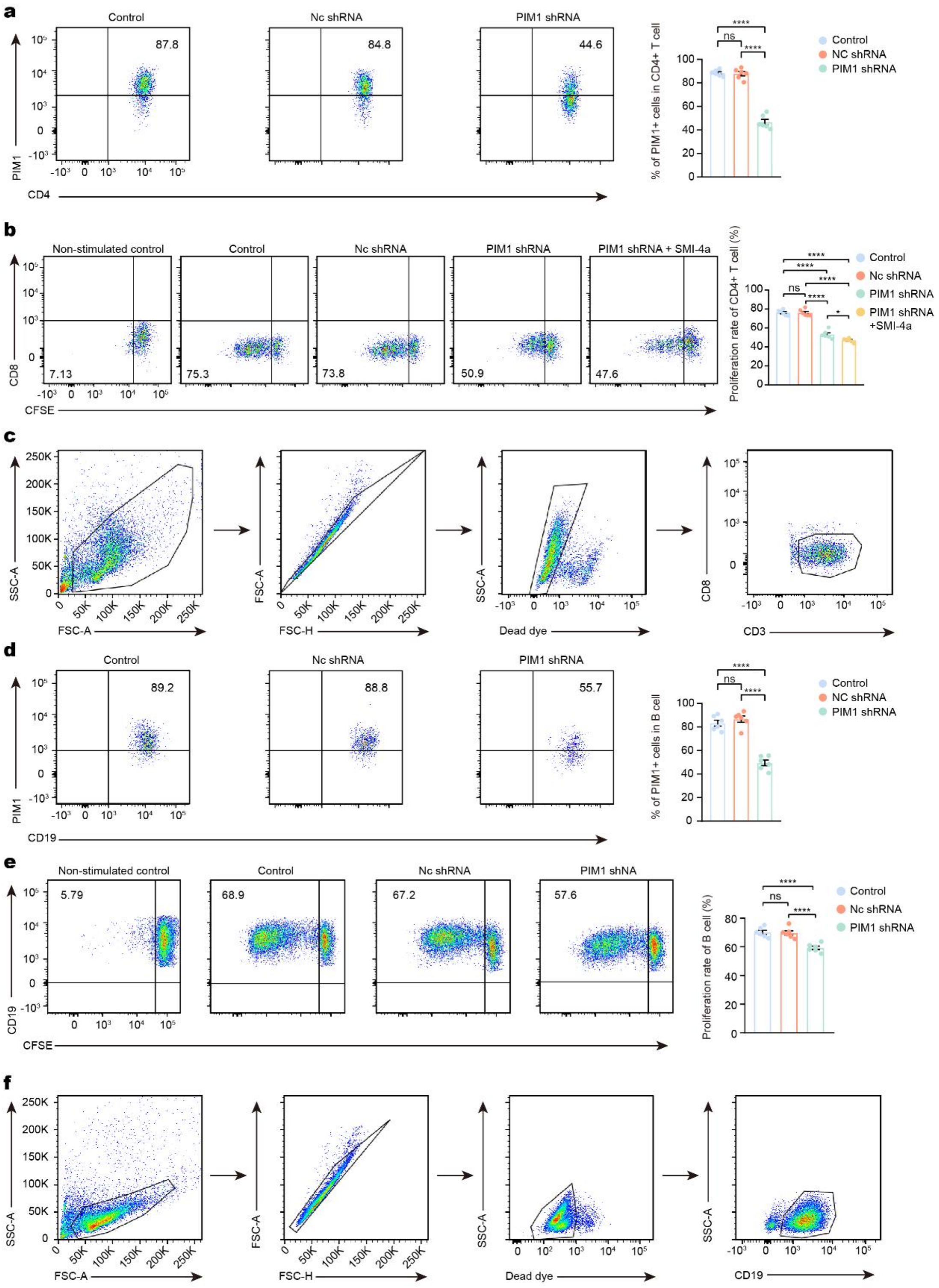

Supplementary Figure 12. PIM1 shRNA inhibited proliferating of CD4⁺ T cells and B cells

(**a**) After treated with PIM1 shRNA or negative control (nc) shRNA, the proportion of PIM1⁺ cells in CD4⁺ T cells was measured by flow cytometry. Data represented as mean ± SEM from six independent experiments. *P*(Control-Nc shRNA) = 0.9773, *P*(Control-PIM1

shRNA) = 2.2E-10, *P*(Nc shRNA-PIM1 shRNA) = 2.6E-10. Significance was determined using one-way ANOVA. ns, no significant differences, ****$P < 0.0001$.

(**b**) After treated with PIM1 shRNA or negative control (nc) shRNA or PIM1 shRNA plus SMI-4a, the proliferating rate of $CD4^+$ T cells (gated on $CD3^+CD8^-$ T cells) measured by flow cytometry. Data represented as mean ± SEM from six independent experiments. *P*(Control-Nc shRNA) = 1.0000, *P*(Control-PIM1 shRNA) = 8.0E-10, *P*(Control-PIM1 shRNA+SMI-4a) = 1.2E-11, *P*(Nc shRNA-PIM1 shRNA) = 8.9E-10, *P*(Nc shRNA-PIM1 shRNA+SMI-4a) = 1.3E-11, *P*(PIM1 shRNA-PIM1 shRNA+SMI-4a) = 0.0233. Significance was determined using one-way ANOVA. ns, no significant differences, *$P < 0.05$, ****$P < 0.0001$.

(**c**) Gating strategy of $CD4^+$ T cells (gated on $CD3^+CD8^-$ T cells) for figure 6h and supplementary figure 11e,12b.

(**d**) After treated with PIM1 shRNA or negative control (nc) shRNA, the proportion of $PIM1^+$ cells in B cells was measured by flow cytometry. Data represented as mean ± SEM from six independent experiments. *P*(Control-Nc shRNA) = 0.6172, *P*(Control-PIM1 shRNA) = 3.0E-07, *P*(Nc shRNA-PIM1 shRNA) = 8.3E-08. Significance was determined using one-way ANOVA. ns, no significant differences, ****$P < 0.0001$.

(**e**) After treated with PIM1 shRNA or negative control (nc) shRNA, the proliferating rate of B cells measured by flow cytometry. Data represented as mean ± SEM from six independent experiments. *P*(Control-Nc shRNA) = 0.9770, *P*(Control-PIM1 shRNA) = 0.0002, *P*(Nc shRNA-PIM1 shRNA) = 0.0002. Significance was determined using one-way ANOVA. ns, no significant differences, *$P < 0.05$, ***$P < 0.001$.

(**f**) Gating strategy of B cells for figure 6i and supplementary figure 12e.

Supplementary Figure 13

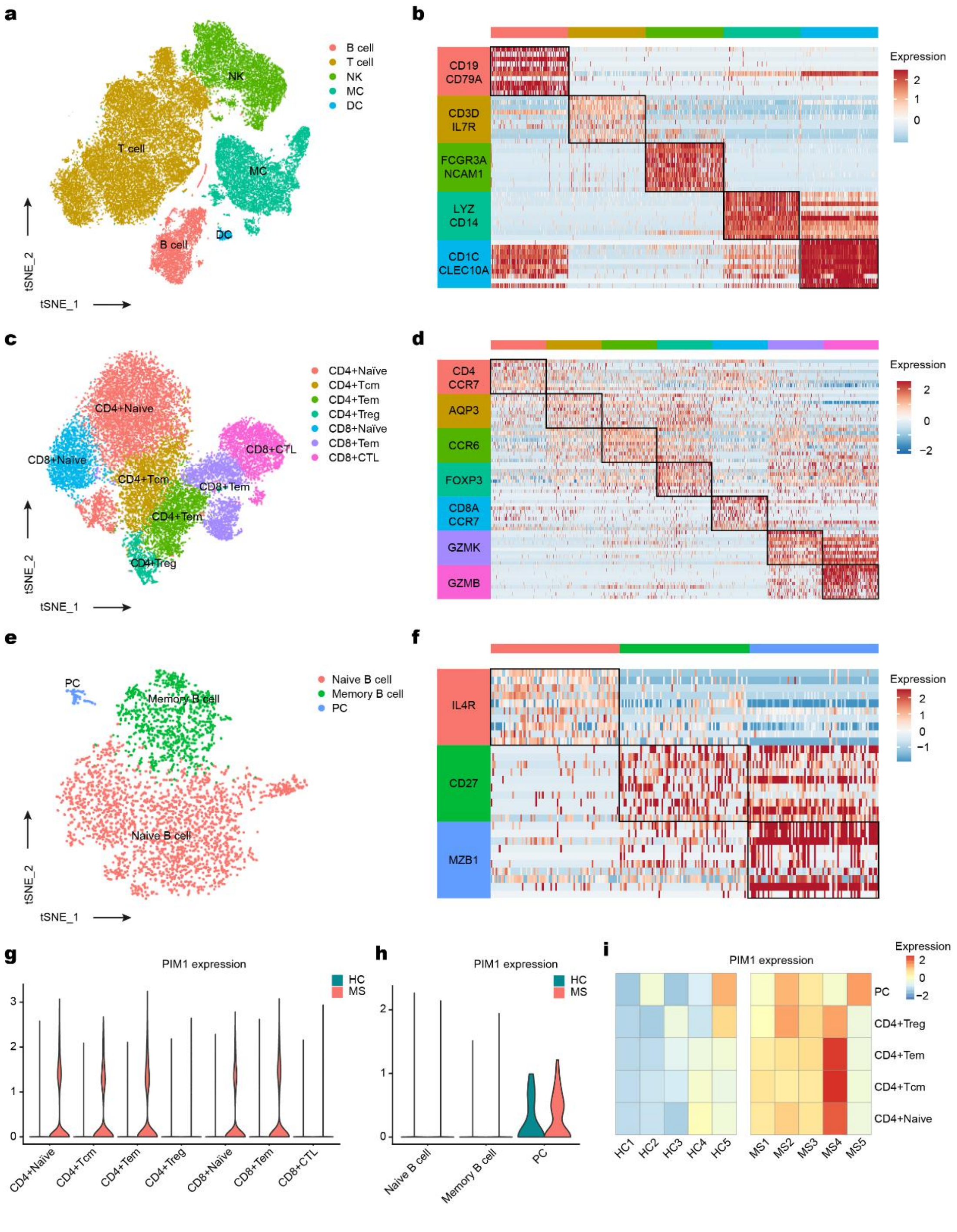

Supplementary Figure 13. Clusters of major immune cell populations, T cell subset and B cell subset, and analysis of PIM1 alterations in MS

(**a**) t-SNE plots of major immune cell types from all healthy controls and MS patients.

(**b**) Heatmap showing scaled expression of discriminative gene sets for each immune cell type from all healthy controls and MS patients.

(**c**) t-SNE plots of T cell subsets from all healthy controls and MS patients.
(**d**) Heatmap showing scaled expression of discriminative gene sets for T cell subsets from all healthy controls and MS patients.
(**e**) t-SNE plots of B cell subsets from all healthy controls and MS patients.
(**f**) Heatmap showing scaled expression of discriminative gene sets for B cell subsets from all healthy controls and MS patients.
(**g**) Violin plots of PIM1 expression in T cell subsets of healthy controls and MS patients.
(**h**) Violin plots of PIM1 expression in B cell subsets of healthy controls and MS patients.
(**i**) Heatmap of the average expression of PIM1 by PCs and CD4 T cell subsets in healthy controls and MS patients.

Supplementary Table

Supplementary Table 1. Clinical and pathological scoring criteria of EAU.

Clinical scoring criteria of EAU	
Grade	Criteria for fundoscopy
0	Normal retina
0.5	Few (<3), small and focal lesions; minimal vasculitis and vitritis
1	Mild vasculitis; multiple, peripheral and focal lesions
2	Retinal edema; severe vasculitis (large size, thick wall, infiltrations); diffuse chorioretinal lesions and/or infiltrations; linear lesions
3	Retinal edema; pattern of linear lesions; large confluent chorioretinal lesions; subretinal hemorrhages
4	Large retinal detachmen
Pathological scoring criteria of EAU	
Grade	Criteria for histopathology
0	Normal retina structure
0.5	Mild inflammatory cell infiltration. No tissue damage
1	Infiltration; retinal folds and focal retinal detachments; few small granulomas in choroid and retina, perivasculitis
2	Moderate infiltration; retinal folds, detachments and focal photoreceptor cell damage; small to medium sized granulomas, perivasculitis and vasculitis
3	Medium to heavy infiltration; extensive retinal folding with detachments, moderate photoreceptor cell damage; medium sized granulomatous lesions; subretinal neovascularization
4	Heavy infiltration; diffuse retinal detachment with serous exudate and subretinal bleeding; extensive photoreceptor cell damage; large granulomatous lesions; subretinal neovascularization

Supplementary Table 2. Number of reads and total genes detected in single-cell RNAseq.

Human sample		
Sample name	Number of reads	Total genes detected
VKH1	392385627	21029
VKH2	490406166	21743
VKH3	321676246	21005
VKH4	315619659	21628
VKH5	329400422	21461
VKH6	328539775	20056
HC1	32450834	18076
HC2	450476544	20687
HC3	331572739	20994
HC4	471824429	21701
HC5	437526554	21718
HC6	455368571	22062
Mice sample		
Sample name	Number of reads	Total genes detected
D0_1	346135428	18128
D0_2	373629351	18179
D7_1	399816989	18281
D7_2	414002815	18920
D14_1	363313196	18790
D14_2	375995953	18816
D21_1	394788267	18079
D21_2	422916905	18346
D7c	412726691	18984
D14c	433444916	19102
D21c	417749658	18441

Supplementary Table 3. Information of HC and VKH sample

Group	Age	Gender	Single-cell RNAseq	Flow cytometry
VKH1	36	female	YES	NO
VKH2	33	female	YES	NO
VKH3	61	female	YES	NO
VKH4	45	male	YES	NO
VKH5	64	female	YES	NO
VKH6	50	female	YES	NO
VKH7	27	female	NO	YES
VKH8	36	male	NO	YES
VKH9	48	male	NO	YES
VKH10	50	male	NO	YES
VKH11	47	female	NO	YES
VKH12	41	female	NO	YES
VKH13	28	male	NO	YES
VKH14	66	female	NO	YES
VKH15	55	male	NO	YES
VKH16	25	female	NO	YES
VKH17	30	female	NO	YES
VKH18	22	female	NO	YES
VKH19	51	male	NO	YES
VKH20	48	male	NO	YES
VKH21	60	male	NO	YES
HC1	25	female	YES	NO
HC2	24	male	YES	NO
HC3	24	male	YES	NO
HC4	27	male	YES	NO
HC5	66	female	YES	NO
HC6	58	male	YES	NO
HC7	52	male	NO	YES
HC8	43	female	NO	YES
HC9	61	male	NO	YES
HC10	39	female	NO	YES
HC11	29	male	NO	YES
HC12	30	female	NO	YES
HC13	44	male	NO	YES
HC14	37	female	NO	YES
HC15	60	male	NO	YES
HC16	33	male	NO	YES
HC17	62	male	NO	YES
HC18	53	female	NO	YES
HC19	42	female	NO	YES

HC20	36	female	NO	YES
HC21	28	female	NO	YES
