# Supplementary material for: Multicellular immune dynamics implicate PIM1 as a potential therapeutic target for uveitis
Source: Nat Commun. 2022 Oct 4;13:5866. doi: 10.1038/s41467-022-33502-7 (PMC9532430; doi:10.1038/s41467-022-33502-7)
Supplement: Supplementary file 2 — Description of Additional Supplementary Files [file 41467_2022_33502_MOESM2_ESM.pdf]

Supplementary Data 1: Supplementary Data 1 shows the differentially expressed genes(DEGs) used in volcano plot.
