# Supplementary material for: Multicellular immune dynamics implicate PIM1 as a potential therapeutic target for uveitis
Source: Nat Commun. 2022 Oct 4;13:5866. doi: 10.1038/s41467-022-33502-7 (PMC9532430; doi:10.1038/s41467-022-33502-7)
Supplement: Supplementary file 4 — Reporting Summary [file 41467_2022_33502_MOESM4_ESM.pdf]

nature portfolio

Corresponding author(s): Xialin Liu, Yingfeng Zheng, Wenru Su

Last updated by author(s): Aug 13, 2022

Reporting Summary

Nature Portfolio wishes to improve the reproducibility of the work that we publish. This form provides structure for consistency and transparency in reporting. For further information on Nature Portfolio policies, see our Editorial Policies and the Editorial Policy Checklist.

Statistics

For all statistical analyses, confirm that the following items are present in the figure legend, table legend, main text, or Methods section.

n/a	Confirmed	
☐	☒	The exact sample size (n) for each experimental group/condition, given as a discrete number and unit of measurement
☐	☒	A statement on whether measurements were taken from distinct samples or whether the same sample was measured repeatedly
☐	☒	The statistical test(s) used AND whether they are one- or two-sided<br>*Only common tests should be described solely by name; describe more complex techniques in the Methods section.*
☒	☐	A description of all covariates tested
☐	☒	A description of any assumptions or corrections, such as tests of normality and adjustment for multiple comparisons
☐	☒	A full description of the statistical parameters including central tendency (e.g. means) or other basic estimates (e.g. regression coefficient) AND variation (e.g. standard deviation) or associated estimates of uncertainty (e.g. confidence intervals)
☐	☒	For null hypothesis testing, the test statistic (e.g. F, t, r) with confidence intervals, effect sizes, degrees of freedom and P value noted<br>*Give P values as exact values whenever suitable.*
☒	☐	For Bayesian analysis, information on the choice of priors and Markov chain Monte Carlo settings
☒	☐	For hierarchical and complex designs, identification of the appropriate level for tests and full reporting of outcomes
☒	☐	Estimates of effect sizes (e.g. Cohen's d, Pearson's r), indicating how they were calculated

Our web collection on statistics for biologists contains articles on many of the points above.

Software and code

Policy information about availability of computer code

Data collection	ZEISS LSM 880(confocal microscopy), BD LSRFortessa (flow cytometry), Illumina NovaSeq6000 (scRNA seq)
Data analysis	Cell Ranger (version 3.0.2), R (version 4.0.0), Seurat (version 3.1), Harmony (version 1.0), ggplot2 (version 3.2.1), pheatmap (version 1.0.12), FlowJo (version 10.0.7), CellRanger vdj pipeline (version 3.0.2), GraphPad Prism (version 8.0.2), ImageJ (version 1.46r)

For manuscripts utilizing custom algorithms or software that are central to the research but not yet described in published literature, software must be made available to editors and reviewers. We strongly encourage code deposition in a community repository (e.g. GitHub). See the Nature Portfolio guidelines for submitting code & software for further information.

Data

Policy information about availability of data

All manuscripts must include a data availability statement. This statement should provide the following information, where applicable:

- Accession codes, unique identifiers, or web links for publicly available datasets
- A description of any restrictions on data availability
- For clinical datasets or third party data, please ensure that the statement adheres to our policy

The source data are provided with this paper. The single-cell sequencing data generated in this study was deposited in the Genome Sequence Archive (GSA) under project number (PRJCA004238) and GSA accession number (mouse data: CRA003777 [https://download.cncb.ac.cn/gsa/CRA003777]; human data: HRA002504 [https://ngdc.cncb.ac.cn/gsa-human/browse/HRA002504]).

Field-specific reporting

Please select the one below that is the best fit for your research. If you are not sure, read the appropriate sections before making your selection.

- [x] Life sciences
- [ ] Behavioural & social sciences
- [ ] Ecological, evolutionary & environmental sciences

For a reference copy of the document with all sections, see nature.com/documents/nr-reporting-summary-flat.pdf

Life sciences study design

All studies must disclose on these points even when the disclosure is negative.

Sample size	Sample sizes were indicated in the legend of each Figure and Supplementary Figure. No statistical methods were used to predetermine sample sizes. Sample size choice was based on previous studies in the field (Wang G, Li X, Li N, et al. 2022. DOI: 10.1016/j.redox.2022.102297 ; ZChen Z, Zhang T, Kam HT, et al. 2021. DOI: 10.1016/j.ebiom.2021.103496; Bing SJ, Shemesh I, Chong WP, et al. 2019. DOI: 10.1016/ j.jaut.2019.02.006; Huang Z, Chen B, Liu X, et al. 2021. DOI: 10.1073/pnas.2023216118; Hu Y, Hu Y, Xiao Y, et al. 2020. DOI: 10.1073/ pnas.2002476117).
Data exclusions	For quality control, our filter criteria were 300-4000 genes and less than 15% of mitochondrial genes. Furthermore, for human sample, we filtered out the cells highly expressing HBB, HBA1, and several light and heavy chain transcripts, which were considered to be RBC-contaminated cell populations. For mouse sample, cells with high expression of Hbb-a1 and Hbb-bs that were recognized as red blood cells were filtered.
Replication	The experimental findings were reliably reproduced, for representative data used for statistical analysis, the number of samples or experiments is described in corresponding figure legends.
Randomization	All samples were randomly allocated into experimental groups.
Blinding	The investigators were blinded to grouped allocation during data collection and analysis

Reporting for specific materials, systems and methods

We require information from authors about some types of materials, experimental systems and methods used in many studies. Here, indicate whether each material, system or method listed is relevant to your study. If you are not sure if a list item applies to your research, read the appropriate section before selecting a response.

Materials & experimental systems

n/a	Involved in the study	
[ ]	[x]	Antibodies
[x]	[ ]	Eukaryotic cell lines
[x]	[ ]	Palaeontology and archaeology
[ ]	[x]	Animals and other organisms
[ ]	[x]	Human research participants
[x]	[ ]	Clinical data
[x]	[ ]	Dual use research of concern

Methods

n/a	Involved in the study	
[x]	[ ]	ChIP-seq
[ ]	[x]	Flow cytometry
[x]	[ ]	MRI-based neuroimaging

Antibodies

Antibodies used	anti-mouse CD90.2 (Thy-1.2) (clone 53-2.1) Super Bright 702 Invitrogen Cat# 67-0902-82, RRID: AB_2717157<br>anti-mouse Foxp3 (clone FJK-16s) FITC Invitrogen Cat# 11-5773-82, RRID: AB_465243<br>anti-mouse CD11c (clone HL3) PE BD Pharmingen Cat# 557401, RRID: AB_396684<br>anti-mouse F4/80 (clone BM8) Brilliant Violet 650™ Biolegend Cat# 123149, RRID: AB_2564589<br>anti-human/mouse CD45R (B220) (clone RA3-6B2) eFluor 506 Biolegend Cat# 69-0452-82, RRID: AB_2637455<br>anti-mouse CD138 (Syndecan-1) (clone 281-2) APC Biolegend Cat# 142505, RRID: AB_10960141<br>anti-mouse CD4 (clone GK1.5) PerCP/Cyanine5.5 Biolegend Cat# 100434, RRID: AB_893324<br>anti-mouse CXCR5 (clone L138D7) FITC Biolegend Cat# 1145519, RRID: AB_2562865<br>anti-mouse PD-1 (clone 29F.1A12) APC/Cyanine7 Biolegend Cat# 135223, RRID: AB_2563522<br>anti-mouse Fas (clone SA367H8) PE/Cyanine7 Biolegend Cat# 152617, RRID: AB_2910313<br>anti-mouse GL7 (clone GL7) APC Biolegend Cat# 144617, RRID: AB_2800674<br>anti-mouse IL-17A (clone TC11-18H10.1) Brilliant Violet 650™ Biolegend Cat# 506930, RRID: AB_2686975<br>anti-mouse IFN-γ (clone XMG1.2) PE Biolegend Cat# 505808; RRID: AB_315402<br>anti-mouse CD25 (clone PC61) PE/Cyanine7 Biolegend Cat# 102016, RRID: AB_312865<br>anti-PIM1 (clone ST0513) NOVUS Cat# NBP2-67528, RRID:AB_2921371

	anti-phospho-FOXO1 (clone Ser256) Invitrogen Cat# PA5-104977, RRID: AB_2816450<br>anti-phospho-AKT1 (clone Ser473) APC Invitrogen Cat# 17-9715-42, RRID: AB_2573310<br>anti-human CD3 (clone UCHT1) Brilliant Violet 785™ Biolegend Cat# 300471, RRID: AB_2687177<br>anti-human CD8a (clone RPA-T8) PE/Cyanine7 Biolegend Cat# 301012, RRID: AB_314130<br>anti-human CD19 (clone HIB19) Brilliant Violet 605™ Biolegend Cat# 302244, RRID: AB_2562015<br>anti-human CD38 (clone HIT2) Brilliant Violet 785™ Biolegend Cat# 303529, RRID: AB_2561368<br>anti-human CD20 (clone 2H7) APC Biolegend Cat# 302309, RRID: AB_314257<br>anti-human CD4 (clone SK3) APC Biolegend Cat# 344613, RRID: AB_2028485<br>anti-rabbit IgG (H+L), F(ab')2 Fragment (Alexa Fluor® 488 Conjugate) secondary antibody, Cell Signaling Technology, Cat# 4412
Validation	All primary antibodies used in this study were validated by the manufacture. Validation data / citations can be found on the manufacture website by searching the antibody catalog number provided in materials and methods section of our manuscript.

Animals and other organisms

Policy information about studies involving animals; ARRIVE guidelines recommended for reporting animal research

Laboratory animals	Wild-type C57BL/6J female mice (6–8–week–old, 18–25 g) were purchased from Medical Lab Animal Center.<br>All mice were housed in specific pathogen-free environment at 21 ± 1 °C and 60 ± 5% humidity, with a 12-h light/dark cycle.<br>Experimental and control animals were bred separately.
Wild animals	The study did not involve wild animals.
Field-collected samples	No field collected samples were used in the study since the information currently provided here is not relevant to this field.
Ethics oversight	Animal experiments in our study were allowed by the Institutional Animal Care Committee (Zhongshan Ophthalmic Center, Sun Yat-Sen University).

Note that full information on the approval of the study protocol must also be provided in the manuscript.

Human research participants

Policy information about studies involving human research participants

Population characteristics	Healthy donors and patients with VKH are 20~70 years old males and females.
Recruitment	Human peripheral blood of healthy donors and patients with VKH were collected from tZhongshan Ophthalmic Center, Sun Yat-sen University, Guangzhou, China.<br>Samples from healthy people showed no history of major diseases and had normal biochemical indicators.<br>Samples from patients with VKH were diagnosed based on disease manifestations and the results of standard coherent optical tomography and indocyanine green fluorescein angiography, according to the Revised Diagnostic Criteria (RDC) of VKH disease.
Ethics oversight	Informed consent was obtained by all participants. All protocols were reviewed and approved by the Medical Ethics Committee of the Guangzhou Zhongshan Ophthalmic Center (ID:2020KYPJ124).

Note that full information on the approval of the study protocol must also be provided in the manuscript.

Flow Cytometry

Plots

Confirm that:

- [x] The axis labels state the marker and fluorochrome used (e.g. CD4-FITC).
- [x] The axis scales are clearly visible. Include numbers along axes only for bottom left plot of group (a 'group' is an analysis of identical markers).
- [x] All plots are contour plots with outliers or pseudocolor plots.
- [x] A numerical value for number of cells or percentage (with statistics) is provided.

Methodology

Sample preparation	Mice: To isolate the cells of cervical draining lymph nodes were harvested from the mice . Cells were filtered through a cell strainer to make single-cell suspensions.<br>Human: we extracted the venous blood samples from all donors using Ficoll-Hypaque solution (GE Healthcare, PA, USA), followed by heparinization of the blood, then processing via standard density gradient centrifugation approaches to obtain PBMCs.<br>After staining with live/dead dye, harvested cells were stained with surface markers and analyzed via flow cytometry (BD LSRFortessa). For intracellular staining, the cells were stimulated with 5 ng/mL of phorbol myristate acetate (Sigma), 1 µg/mL brefeldin A (Sigma), and 500 ng/mL ionomycin (Sigma) at 37°C for 4 h. Harvested cells were fixed, permeabilized, stained with antibodies, and analyzed via flow cytometry.

Instrument	BD LSRFortessa
Software	FlowJo (version 10.0.7)
Cell population abundance	Only cell compositions of PBMCs and cervical draining lymph nodes are studied here.
Gating strategy	Cells were first gated in intact cells using FSC/SCC, doublets were excluded using FSC-A vs FSC-H, cells were then gated on live cells using LIVE/DEAD Fixable Yellow Dead Cell Stain Kit, followed by cell type specific gating using fluorescently labeled antibodies

☒ Tick this box to confirm that a figure exemplifying the gating strategy is provided in the Supplementary Information.
